# Structure adaptation in Omicron SARS-CoV-2/hACE2: Biophysical origins of evolutionary driving forces

**DOI:** 10.1016/j.bpj.2023.09.003

**Published:** 2023-09-16

**Authors:** Ya-Wen Hsiao, David J. Bray, Tseden Taddese, Guadalupe Jiménez-Serratos, Jason Crain

**Affiliations:** 1 The Hartree Centre, STFC Daresbury Laboratory, Warrington, United Kingdom; 2 Scientific Computing Department, STFC Daresbury Laboratory, Warrington, United Kingdom; 3 IBM Research Europe, Hartree Centre, Warrington, United Kingdom; 4 Department of Biochemistry, University of Oxford, Oxford, United Kingdom

## Abstract

Since its emergence, the COVID-19 threat has been sustained by a series of transmission waves initiated by new variants of the SARS-CoV-2 virus. Some of these arise with higher transmissivity and/or increased disease severity. Here, we use molecular dynamics simulations to examine the modulation of the fundamental interactions between the receptor binding domain (RBD) of the spike glycoprotein and the host cell receptor (human angiotensin-converting enzyme 2 [hACE2]) arising from Omicron variant mutations (BA.1 and BA.2) relative to the original wild-type strain. Our key findings are that glycans play a vital role at the RBD···hACE2 interface for the Omicrons, and the interplay between glycans and sequence mutations leads to enhanced binding. We find significant structural differences in the complexes, which overall bring the spike protein and its receptor into closer proximity. These are consistent with and attributed to the higher positive charge on the RBD conferred by BA.1 and BA.2 mutations relative to the wild-type. However, further differences between subvariants BA.1 and BA.2 (which have equivalent RBD charges) are also evident: mutations reduce interdomain interactions between the up chain and its clockwise neighbor chain in particular for the latter, resulting in enhanced flexibility for BA.2. Consequently, we see occurrence of additional close contacts in one replica of BA.2, which include binding to hACE2 by a second RBD in addition to the up chain. Although this motif is not seen in BA.1, we find that the Omicrons can directly/indirectly bind a down-RBD to hACE2 through glycans: the role of the glycan on N90 of hACE2 switches from inhibiting to facilitating the binding to Omicron spike protein via glycan-protein lateral interactions. These structural and electrostatic differences offer further insight into the mechanisms by which viral mutations modulate host cell binding and provide a biophysical basis for evolutionary driving forces.

## Significance

The evolution of the SARS-CoV-2 virus will determine the future path of the pandemic. Consequently, it is critical to develop insight into the nature of mutations at the molecular scale and to understand their biophysical role in conferring evolutionary advantage. Using molecular simulations, we examine here the structure of the SARS-CoV-2 viral spike protein in contact with its human receptor target. We establish, using molecular simulation, how the mutations in the Omicron variants lead to altered structural configurations in the spike-receptor complex relative to the original wild-type (Wuhan) strain and within two Omicron subvariants. We interpret these findings in terms of basic biophysical properties such as electric charge and flexibility modified by the mutated protein sequences.

## Introduction

Coronavirus disease 2019 (COVID-19) has been responsible for around 7 million deaths and over 760 million cases worldwide over the course of the pandemic and it remains a significant global health threat. It is caused by the severe acute respiratory syndrome coronavirus 2 (SARS-CoV-2)—a single-stranded RNA-enveloped virus and the seventh known coronavirus to infect humans. Since its wild-type (WT) strain was first discovered in Wuhan, China, the virus has undergone continuous mutation leading to a succession of so-called variants of concern (VOCs). Designated by the World Health Organization as Alpha to Delta, and Omicron, these VOCs occur with increasing transmission rate and variable pathogenicity.

In common with other coronaviruses, the virion surface consists of spike (S) glycoproteins comprising two (S1 and S2) subunits. The S2 subunit embeds the spike in the viral envelope and mediates viral fusion with the host cell membrane upon protease activation. The S1 subunit is involved in recognition and binding to the peptidase domain of the host receptor angiotensin-converting enzyme 2 (hACE2). It includes a trimer of N-terminal domains (NTDs) and receptor binding domains (RBDs), each of which exists in either of two discrete up or down conformations in the prefusion state. Only in the up conformation is the receptor binding site exposed. Typically, in the active conformation one of the three RBD domains is in the up state with the other two in the down position (1).

The, still dominant, Omicron strain is unique among known variants as it exhibits a much larger number of point mutations than any of its predecessors—more than 30 on the S-protein, of which over half are found in the RBD. Since vaccine strategies rely heavily on the spike sequence, the emergence of such hypermutated strains threatens to weaken the neutralizing activity of vaccine-induced antibodies. Indeed, there is evidence that Omicron subvariants BA.1, BA.2 exhibit immune escape among double-vaccinated hosts, and require a third dose to induce the neutralizing immune response (2,3).

The molecular mechanisms by which the SARS-CoV-2 RBD recognizes and associates with hACE2 are therefore fundamental to the infection process. They are also essential for elucidating the biophysical consequences of mutation and, thereby, the molecular basis for evolutionary driving forces. For example, relative to WT, the net positive charge on each RBD increases by two and three for Delta and Omicron variants, respectively. This increase in charge is accompanied both by higher infection rate and reduced potency of neutralizing antibodies (4,5,6,7,8,9,10,11,12). Both effects are consistent with evolutionary pressure toward RBD mutations which increase the net positive charge: firstly, as hACE2 is a charge-negative entity (13), the electrostatic interaction is increased; and secondly, the electrostatic surfaces of neutralizing antibodies reveal that most antibodies have positively charged RBD-recognition domains. However, so far reports generally have focused on binding free energy differences. Most of these were evaluated based on the binding structure of RBD+hACE2 for WT. Structural changes in the RBD+hACE2 complex arising from Omicron-lineage mutations have been less explored in comparison.

The effects of the individual point mutations on RBD with respect to infectivity have also been another focus of recent studies. For example, N501Y, the RBD mutation in the Alpha variant enhanced the binding affinity to hACE2 (14,15); E484K in Beta and Gamma variants reduced the neutralization activity of human sera (16); L452R in Delta and BA.2 variants have been shown to strengthen fusogenicity and infectivity by enhancing the cleavage of spike protein; and K417N removes the interfacial salt bridge between the RBD and neutralizing antibody CB6 (11). Questions naturally arise concerning whether there are more ways to associate the high infectivity and the many point mutations on each RBD for BA.1/BA.2.

Fifteen and sixteen point mutations occur on each RBD of BA.1 and BA.2, respectively, and affect the interaction between RBD and hACE2. The mutations in the RBD of BA.1 and BA.2 contribute to an increase of the net positive charge by three compared with WT. A stronger electrostatic attraction of the RBD+hACE2 complex is thus expected for Omicron than WT. However, this leaves an obvious question as to why BA.2 is more infectious than BA.1 (17) as both have the same net charge at the RBD. Thus, the specifics of the localized electrostatic landscape must matter (i.e., interactions involving specific mutations) in binding the complex system.

Another key feature of the S-protein is its extensive glycosylation. Each monomer S-protein displays 22 potential N-linked and four O-linked glycosylation sites (18,19). Cryoelectron microscopy provides evidence for the existence of 17 N-glycans on 22 potential sites in the SARS-CoV-2 S-protein (1,20), while the remaining five sites are found unoccupied (19). These glycans shield about 40% of the trimeric S-protein surface (21), as well as other epitopes from cells and antibody recognition, and enable the coronavirus to evade both the innate and adaptive immune responses (1,22). Thus, glycosylation plays a vital role in the pathogenesis of viruses such as coronavirus (18,23,24). In addition, receptor hACE2 is also heavily glycosylated, and the glycan on N90 of hACE2 ($\bar{N}$90_hACE2_) was found to be able to interfere with the binding between hACE2 and S-protein (25,26), whereas $\bar{N}$322_hACE2_ interacts tightly with the RBD of the hACE2-bound S-protein and strengthens the complex (26). Therefore, it is evident that glycans, either on the S-protein or on hACE2, are an important factor for the formation of S+hACE2 complex.

Herein, we use molecular dynamics (MD) simulations to investigate the structural and biophysical mechanisms by which mutations in SARS-CoV-2 modulate receptor recognition and binding. We focus on the Omicron variant BA.1, and its sublineage BA.2 in complex with hACE2, and compared these with that of the WT. In addition to elucidating the effects of mutations in the binding of this complex system, we also examine the role of the glycans at the interface in forming S+hACE2 complex.

## Methods

**Figure 1:**
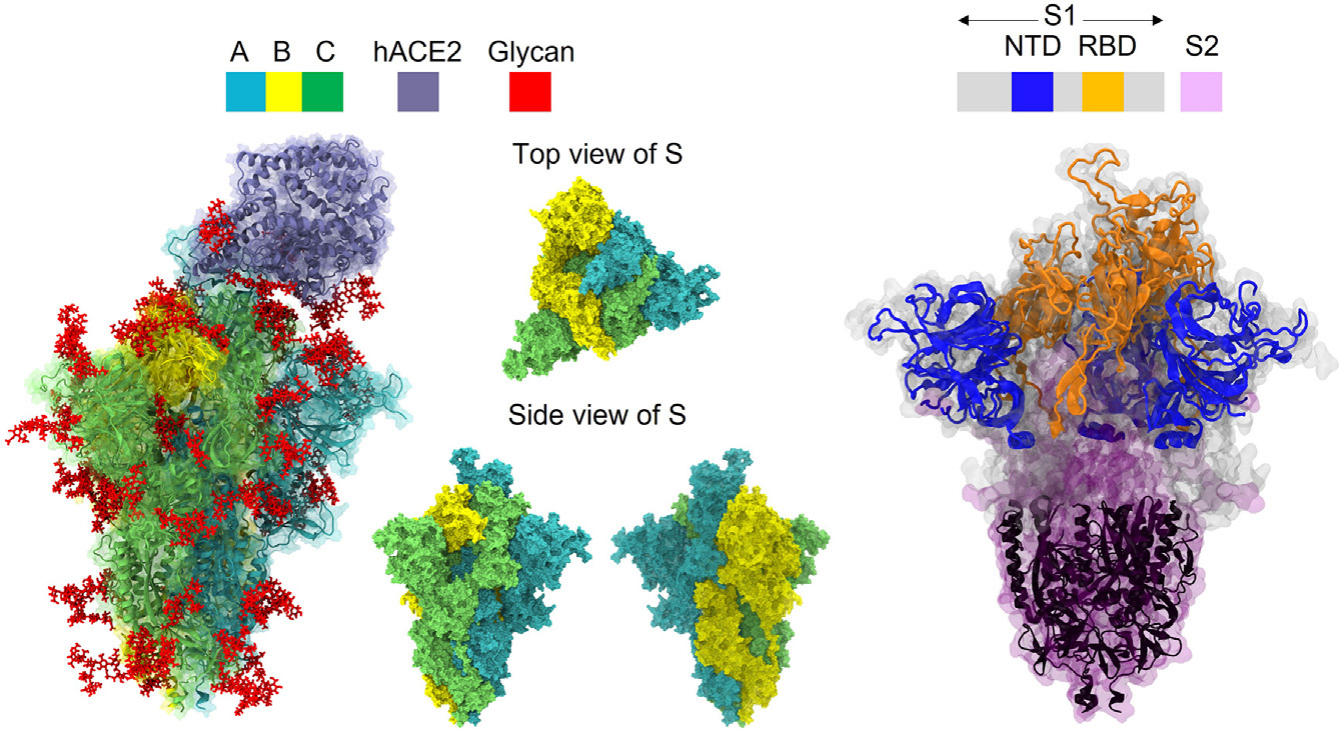
Representations of the quaternary structure of the spike protein and hACE2 highlighting the S trimer chains A–C, key domain structure—NTD (residues 16–291) and RBD (residues 330–530)—and bound glycans.

The structures for hACE2 and WT S-protein in the up state were modeled using PDB: 6VW1 (27) and PDB: 6VSB (20), respectively. The spatial arrangement of the three chains in the S-protein is shown in Fig. 1. The color code of each S-protein chain is followed throughout this paper: chain A is the up chain (*cyan*), chain B is the counterclockwise chain (*yellow*), and chain C is the clockwise neighbor chain (*lime*). We obtained these from the CHARMM-GUI (28) Archive–COVID-19 Protein Library, which arranged the position of hACE2 and S-protein, as given by Woo et al. (29). Here, Woo et al. (29) modified the amino acid sequence of PDB: 6VSB to match the WT and had the remaining missing atoms of S-protein reconstructed.

Using the sequence of the WT S-protein as the template, we generated the Omicron subvariants BA.1 (30) and BA.2 (31) by introducing the corresponding sequence changes via the VMD plugin psfgen (32,33). Glycosylation of hACE2 and S-protein—as proposed by Shajahan et al. (34) (5 N-glycans) and Woo et al. (29) (1 O- and 19 N-glycans), respectively—was introduced using the Glycan Modeler within CHARMM-GUI (35).

Residues 19 to 614 of a monomer hACE2 and residues 1 to 1146 of trimeric S-protein were then solvated in a cubic box of TIP3P water (36) with 0.15 M NaCl and charge neutralized with additional Na ions (i.e., H_2_O/Na/Cl of 495,442/1448/1406, 495,060/1426/1405, and 495,204/1426/1405 molecules, respectively, for WT, BA.1, and BA.2). Hence, resulting in initial box size of 255 Å × 255 Å× 255 Å (where the combined S+hACE2 structure takes up at most 234 Å × 170 Å × 158 Å).

All simulations were carried out using software NAMD 2.12 (37) and the CHARMM36 force field (38,39). First, the initial system was energy minimized using steepest descent to remove atom overlap and then the solvent was relaxed by running an MD simulation where the backbone atoms were restrained with a force constant of 5 kcal mol^−1^ Å^−2^ for a simulated 10 ns duration. Afterward, an unrestrained MD production run was performed. For each MD run, a 2 fs timestep was used with periodic boundary conditions and NPT ensemble (at 310 K and 1.01325 bar conditions) applied using a Langevin thermostat (1 ps^−1^ damping) and Nosé-Hoover Langevin piston barostat (50 fs period and 25 fs decay). The nonbonded interaction cutoff was set at 12 Å and the switch distance at 10 Å. Bonds involving hydrogen were made rigid using the SHAKE algorithm (40). Electrostatics were calculated using the particle mesh Ewald (PME) approach (41) (1 Å grid spacing and 6 PME interpolation order). To avoid translation and rotation, and to limit swaying motions, part of the S2 subunit (over residues 701–729, 787–824, 866–939, and 1035–1146 on all three chains) was anchored via restraints on the backbone atoms using a 20 kcal mol^−1^ Å^−2^ force constant.

Full details on the analyses can be found in the supporting material. We calculated the center of mass (COM) of a chain domain and used these to derive the distance between domains α and β, $d_{\beta }^{\alpha }$. The RBD opening angle of chain A (as used by Fallon et al. (42)), $\theta_{\text{RBD}-A}$, is defined between the COMs of residues 338–517 of chain A, 324–327 and 538–585 of chain A, and 747–755 of chain C. By rotational symmetry, $\theta_{\text{RBD}-C}$ is defined the same as above but replacing A → C and C → B.

Two replicas were run for each system: one is 500 ns in length (RS) and the other extends to 1 *μ*s (RL). For BA.2 an additional replica was run where the restraints on the lower S2 unit were removed. In total, seven copies of simulations were performed. In the simulations by around 600 ns (RL) and 400 ns (RS) the measured COM distances plateaued (Figs. S1 and S2) and we considered the structures equilibrated. PDB files from the end of the RL simulations are included as part of the supporting material, and Table S1 provides the segment names of the systems for readers to view the protein-glycan structures.

## Results and discussion

### Two types of S+hACE2 conformations

Our results show two conformations for the S+hACE2 binding: one, an interface solely via RBD-A···hACE2 contact which we see for WT replicas (RBD-A in its up state); two, interfacing that involves both RBD-A···hACE2 and RBD-C···glycan(s)···hACE2 contacts, which we see for Omicron variants BA.1 and BA.2 (i.e., contact involving a down chain of S). Furthermore, for the RL replica of BA.2, the system reveals the possibility of forming direct contacts between RBD-C and hACE2. We do not observe contact involving RBD-B in any replica.

We compared the up state structures of the three (sub)variants by measuring the opening angle, $\theta_{\text{RBD}-A}$, of the S-protein. For both replicas, we find that the time averaged value for $\theta_{\text{RBD}-A}$ ranked WT > BA.2, BA.1 (Table 1; Fig. S3, *a* and *c*). We also measured the opening angle $\theta_{\text{RBD}-C}$ to check whether chain C becomes open to bind hACE2 (Table 1; Fig. S3, *b* and *d*). We find that the Omicrons have a larger $\theta_{\text{RBD}-C}$ than WT (i.e., RBD-C is more open for the Omicrons), but are much less open than RBD-A, where $\theta_{\text{RBD}-A}$ >75° compared with $\theta_{\text{RBD}-C}$ <60° (i.e., RBD-C is never observed in the fully open state). For completeness, we measured $\theta_{\text{RBD}-B}$ and found that $\theta_{\text{RBD}-B}$ is typically less than 60° (except for WT replica RL which was <71°). This result implies that RBD-B of BA.1/BA.2 is also closed.

**Table 1:** Average (with SD given) RBD opening angles (°); and COM distance (Å) between each RBD and hACE2

Variant	Replica	$\theta_{\text{RBD}-A}$	$\theta_{\text{RBD}-B}$	$\theta_{\text{RBD}-C}$	$d_{\text{hACE}2}^{\text{RBD}-A}$	$d_{\text{hACE}2}^{\text{RBD}-B}$	$d_{\text{hACE}2}^{\text{RBD}-C}$
WT	RL	88.7 ± 1.8	70.5 ± 1.7	45.2 ± 1.1	48.8 ± 0.5	77.6 ± 2.3	61.0 ± 1.8
WT	RS	91.0 ± 2.2	58.1 ± 1.1	46.9 ± 1.0	51.1 ± 0.5	82.1 ± 1.4	60.8 ± 1.1
BA.1	RL	75.1 ± 1.7	52.6 ± 1.1	55.8 ± 1.4	50.3 ± 0.7	76.3 ± 1.5	56.2 ± 1.6
BA.1	RS	83.8 ± 1.3	52.5 ± 1.2	55.0 ± 1.3	48.9 ± 0.4	77.3 ± 1.3	55.0 ± 1.0
BA.2	RL	83.1 ± 1.4	52.2 ± 1.7	58.9 ± 1.8	46.9 ± 0.5	72.7 ± 1.0	51.0 ± 0.4
BA.2	RS	84.9 ± 1.9	48.1 ± 1.2	53.3 ± 1.2	47.1 ± 0.4	80.2 ± 1.2	55.5 ± 0.5
BA.2	no restraints	85.0 ± 1.6	55.1 ± 1.1	52.8 ± 0.9	48.1 ± 0.5	77.8 ± 0.9	56.3 ± 0.5

**Figure 2:**
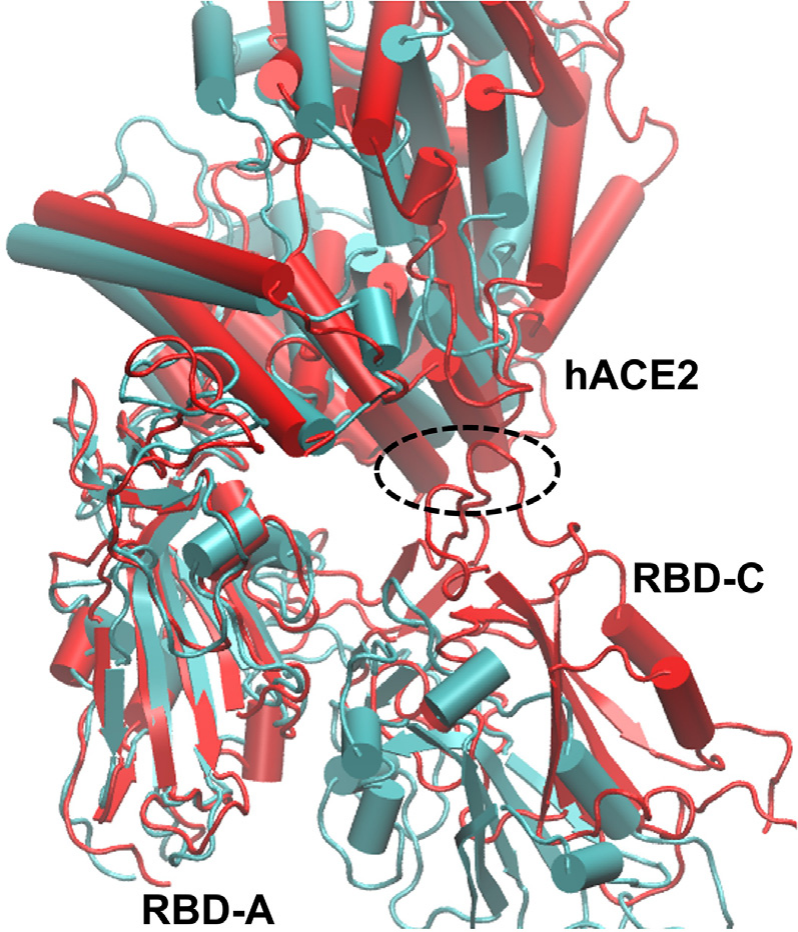
From RL: S+hACE2 complexes involving WT (*cyan*) and BA.2 (*red*), aligned with RBD-A, it is clear that RBD-C of BA.2 is drawn closer to hACE2 (with interaction at the location of the *dashed circle*).

To understand why $\theta_{\text{RBD}-A}$ varies, we investigated the COM distances $d_{\text{hACE}2}^{\text{RBD}-\alpha }$ to find out how the RBD of each individual chain α positions in relation to hACE2, and saw that the Omicrons have smaller $d_{\text{hACE}2}^{\text{RBD}-C}$ (Table 1; Figs. S1 and S2). In both the RL and RS replicas $d_{\text{hACE}2}^{\text{RBD}-B}$ takes the largest value, indicating that RBD-B plays insignificant role in the binding to the hACE2. Typically, $d_{\text{hACE}2}^{\text{RBD}-A}$ is the smallest, with similar values for WT and BA.1 (within ≈2 Å) and slightly smaller values for BA.2 (within ≈4 Å). On the other hand, $d_{\text{hACE}2}^{\text{RBD}-C}$ follows the trend WT > BA.1 > BA.2, with differences of around 5 Å for BA.1 and 5–10 Å for BA.2 compared with WT. This substantial shortening of $d_{\text{hACE}2}^{\text{RBD}-C}$ increases the extent of RBD-C interaction with hACE2 (see Fig. 2), and contributes to the smaller $\theta_{\text{RBD}-A}$ of BA.1/BA.2. As a side note, we confirmed that the effects on the RBD···hACE2 structure of imposing restraints on the S2 unit are small, as evidenced by the similar $\theta_{\text{RBD}-\alpha }$ and $d_{\text{hACE}2}^{\text{RBD}-\alpha }$ values obtained for the *no restraints* replica compared with the other BA.2 replicas.

From visual inspection, we notice the presence of glycans at the interface between RBD-C and hACE2, and show in the text below that the interplay of the Omicron mutations and glycans at this interface is key to modulating $d_{\text{hACE}2}^{\text{RBD}-C}$. To better understand what drives the S+hACE2 complex into the two observed conformations described we performed a detailed study into the contact interactions. Contact comes in two forms: direct interaction between amino acids and indirect protein contact through the selected glycans attached to S-protein and hACE2. In the next sections we look at each in turn.

### Direct interaction between S-protein and hACE2

From our simulation, we identified the contacts between RBD-A and hACE2. One might assume that the positively charged mutations on the RBD enhance the RBD-A binding with hACE2. However, both RL and RS, showed that, more likely, the mutations change the contact partners or probabilities on hACE2, as summarized in Tables 2 and S2, where we see the variation in key contacts RBD-A···hACE2 involving mutated residues. A breakdown list of the contact pairs is provided in Table S3. Mutations involving polar turning into hydrophobic residues S371 L/F, S373P, S375F, and T376A (for BA.2) are located adjacent to neighboring RBD and do not form contact to hACE2.

**Table 2:** The most probable contact pairing between key mutated residues of RBD-A and hACE2 with percent occurrence given in brackets

Residue	WT	BA.1	BA.2
RL			
K417**N**	D30 (90%)	**–**	**–**
N440**K**	–	**E329 (27%)**	**E329 (90%)**
S477**N**	T20 (7%)	**Q24 (3%)**	**S19 (13%)/T20 (10%)**
T478**K**	Q24 (7%)	**Y83/P84 (2%)**	**Q24 (9%)/P84 (6%)**
E484**A**	K31 (17%)	**Q76/L79 (37%)**	**L79 (87%)**
Q493**R**	E35 (77%)/K31 (60%)	**D38/H34 (100%)/K353 (95%)**	**D38/H34 (99%)/K353 (77%)**
G496**S**	K353 (23%)	**K353 (77%)**	–
Q498**R**	Y41 (81%)/Q42 (41%)	**Y41 (94%)/Q42 (57%)**	**Y41 (90%)/Q42 (37%)**
N501**Y**	K353/D355 (100%)/Y41 (86%)	**K353/D355 (100%)/Y41 (77%)**	**K353/D355 (100%)/Y41 (30%)**
Y505**H**	K353/R393 (99%)/E37 (100%)	**G354/K353 (98%)**	**A386/A387 (98%)/K353 (63%)**
D405**N**	A387 (3%)	–	**A387 (71%)**
R408**S**	A387 (3%)	–	**–**
RS			
K417**N**	D30 (47%)/H34 (33%)	**H34 (22%)**	**–**
N440**K**	–	**–**	**E329 (74%)**
S477**N**	T20 (1%)	**S19 (75%)/Q24 (35%)**	**–**
T478**K**	Q24 (18%)	**–**	**–**
E484**A**	K31 (93%)	**–**	**–**
Q493**R**	H34 (50%)/E35 (46%)/K31 (43%)	**H34 (93%)/E35 (97%)/D38 (35%)**	**D38 (93%)/E35 (91%)/H34 (86%)/K353 (14%)**
G496**S**	–	**K353 (67%)/D38 (65%)**	–
Q498**R**	K353 (15%)/Y41 (1%)	**D38 (99%)/Y41 (86%)/Q42 (84%)**	**Y41 (84%)/Q42 (63%)/L45 (19%)**
N501**Y**	K353 (82%)/Y41 (68%)/D355 (18%)	**K353 (100%)/Y41 (76%)/D355 (23%)**	**K353 (99%)/D355 (84%)/Y41 (72%)**
Y505**H**	K353 (95%)/G354 (24%)	**K353 (99%)/G354 (81%)/T27 (21%)**	**K353 (95%)/G354 (72%)/A387 (59%)/E37 (23%)/A386 (19%)**
D405**N**	–	–	**A387 (36%)**
R408**S**	–	–	**–**

Entries in bold indicate contact involving the mutated residue.

**Figure 3:**
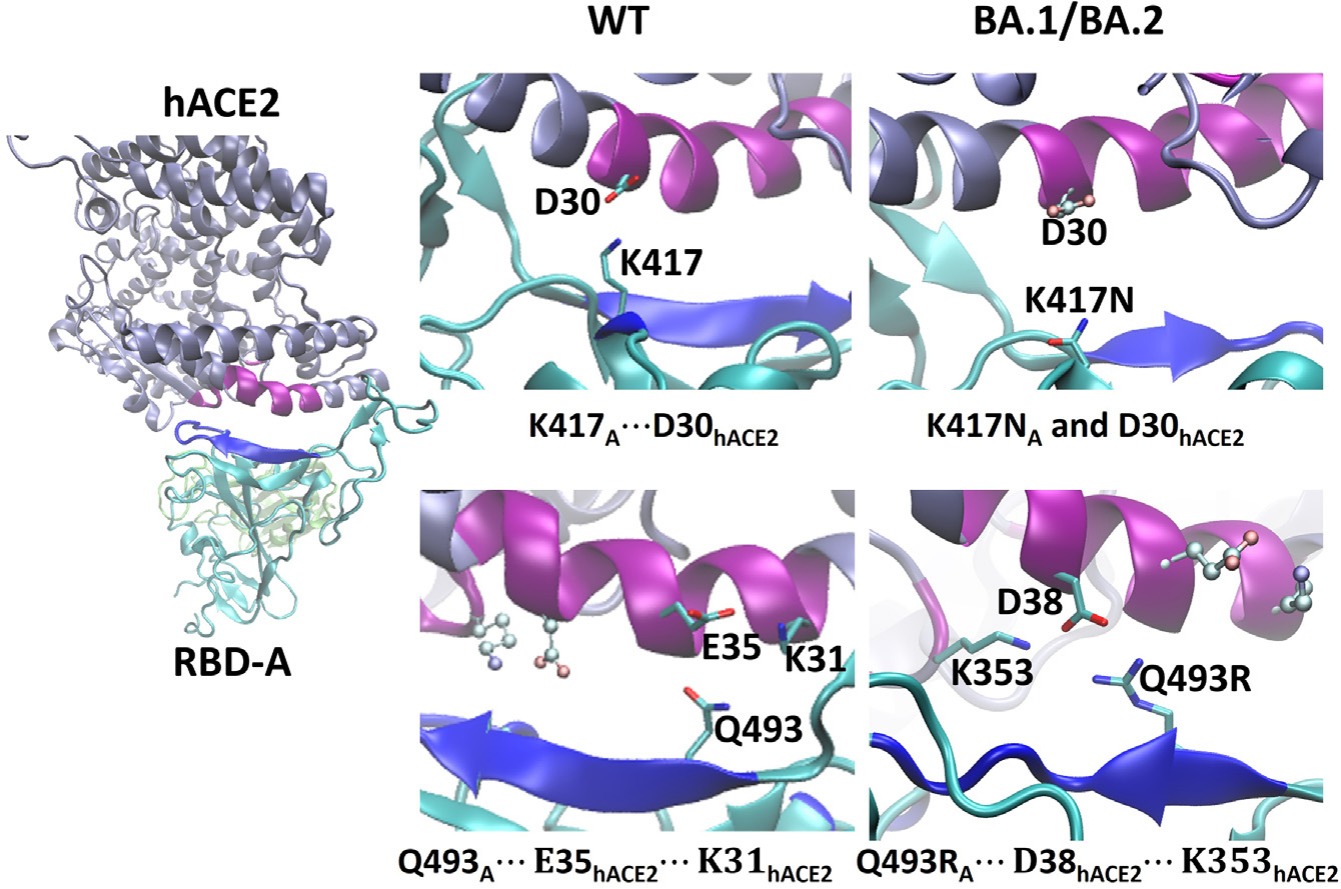
BA.2 (RL): changes in charged contacts between hACE2 and RBD-A (see Table 2): K417_A_ (WT)···D30_hACE2_ (*top center*) was abolished after mutation of K417N (*top right*, where D30_hACE2_ are depicted in pale ball-and-stick); lower row, Q493_A_···E35_hACE2_···K31_hACE2_ (*bottom center*, where D38_hACE2_ and K353_hACE2_ are in pale ball-and-stick) changed to Q493R_A_···D38_hACE2_···K353_hACE2_ (*bottom right*, where E35_hACE2_ and K31_hACE2_ are in pale ball-and-stick).

Tables 2 and S2 also show that the RBD-A···hACE2 contacts are rather dynamic, since there can be more than one residue with high probability of making close contact. Experimental assays and protein structure analysis by Bhattacharjee et al. (43) showed that hACE2 interfacial residues can bind to multiple S-protein residues; for example, K353_hACE2_ can interact with as many as six S-protein residues: Y505, N501, G496, Q498, G502, and Y495. Here, we see that K353_hACE2_ interacts with G496/G496S, N501/N501Y, Y505/Y505H, and Q493R. The seemingly same-charge contact Q493R_A_···K353_hACE2_ of BA.1/BA.2 is due to the bidentate carboxylate of D38_hACE2_ binding to both residues and in turn drawing them close as depicted in Fig. 3. This also demonstrates a change of contact partners from WT (Q493_A_···E35_hACE2_···K31_hACE2_) to BA.1/BA.2 (Q493R_A_···D38_hACE2_···K353_hACE2_).

The mutations D405N and R408S are unique to BA.2 as they are not present in BA.1. D405N eliminates one negative charge and R408S eliminates one positive charge, making the net charge on the RBDs the same for BA.1 and BA.2. However, while D405N_A_ contributes to the interaction with hACE2 (Tables 2 and S2), R408S_A_ does not. This is consistent with the finding of Li et al. (44), who obtained similar binding affinities for R408S mutation in BA.1.1 and the S408R reverse mutation in BA.2, and concluded that the R408S mutation contributes very limited RBD binding to hACE2. The full RBD-A···hACE2 contact probabilities can be seen in the left column of Fig. S4. We also evaluated the difference in the probability of contact for BA.1/BA.2 against WT, as depicted in Figs. S5 and S6, and found that the peak probabilities appear in similar residue neighborhoods when comparing RS with RL, albeit with different magnitudes.

**Figure 4:**
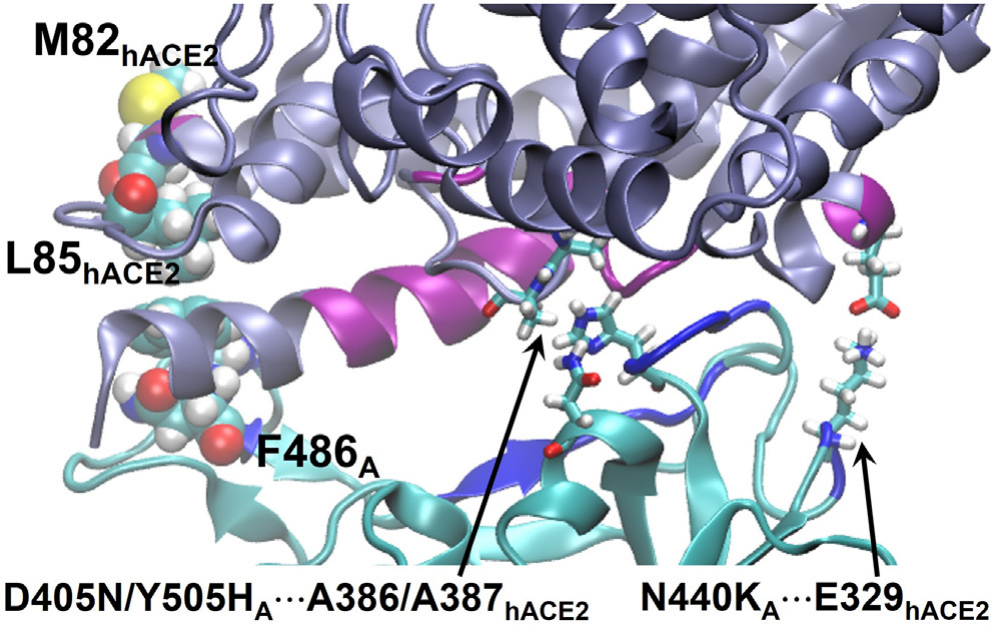
BA.2 (RL): hydrophobic contacts F486_A_···L85_hACE2_, D405N/Y505H_A_···A386/A387_hACE2_, and charged contact N440K_A_···E329_hACE2._

In detail, we find that contacts involving G496S_A_ for BA.1 and D405N_A_, N440K_A_ for BA.2 increase in occurrence relative to WT, with values of 54/88%, 71/38%, and 91/74%, for the RL/RS replicas, respectively. The increase of contact for G496S—a unique mutation for BA.1—is mainly through G496S_A_···K353_hACE2_, whereas the charged-to-polar mutation D405N_A_ of BA.2 engages with a hydrophobic contact A387_hACE2_. Although N440K_A_ is common to BA.1 and BA.2, it interacts more with E329_hACE2_ for BA.2 (Fig. 4) than BA.1, where it is largely solvated. A probability decrease at the mutation site K417N_A_ is seen for both BA.1 and BA.2: K417N_A_ results in the loss of a salt bridge to D30_hACE2_. This observation is consistent with Luan and Huynh (11), who suggest that this mutation may be for eluding antibodies. Differences in interactions involving Q498R_A_ (and neighboring G502_A_) are less clear cut, where we see an increase for Omicron RS replicas but not for the RL replicas compared with WT. This can be explained by the reference values of WT—i.e., only for the RS is there little presence of Q498_A_···Y41_hACE2_ (see Table 2)—whereas this contact is substantially maintained for the Omicron replicas.

We next evaluated the probability of hACE2 residues being in contact with RBD-A to see whether the binding regions on hACE2 changed between variants (shown in the right column of Fig. S4). The differences in probability of contact between the Omicrons and WT are also evaluated in Figs. S7 and S8. The overall binding residue distributions of RL and RS are similar, albeit with different individual probabilities, and it can be seen from the right column of Fig. S4 that the total contact probability is lower in the RS replicas, in particular for WT: when comparing WT with the Omicrons, we see 20% and 46% higher contact probability for BA.1 and BA.2, respectively, reflecting well the larger $d_{\text{hACE}2}^{\text{RBD}-A}$ of WT. We find that, for RL, the total probability of contact is roughly the same among all variants. When comparing between BA.1 and BA.2 in RL, the residues of contact on hACE2 differ mainly at E329_hACE2_ where BA.2 establishes close contact using N440K_A_ (a similar picture is seen for RS, see Tables 2 and S3). Besides E329_hACE2_, for RS, hACE2 forms more charged/polar contacts with BA.2 than BA.1 via N330_hACE2_, D355_hACE2_, and R357_hACE2_. The decrease at R393_hACE2_ in RL for both BA.1 and BA.2 (Fig. S7) does not appear in RS (Fig. S8). This is not because BA.1/BA.2 in RS form contact between RBD-A and R393_hACE2_, but because of WT: while WT forms substantial contact Y505_A_···R393_hACE2_ in RL, none is seen in RS due to longer distance in between. R393_hACE2_ may not be involved in binding to RBD-A of BA.1/BA.2, as we do not see it in either RL or RS. Furthermore, for RL, there is a decrease in contact probability (WT → Omicron) for Y505_A_···E37_hACE2_ and an increase for Q493R_A_···D38_hACE2_, which is caused by Y505_A_ of WT mutating to Y505H_A_ in BA.1/BA.2, and by a salt bridge Q493R_A_···D38_hACE2_ forming in preference to Q493R_A_···E37_hACE2_. This preference to form a salt bridge, seen in RS as well, was also found by Lupala et al. (45) in the case of mutation Q493K (rather than Q493R here). Both RL and RS show higher contact probability between hACE2 and RBD-A of BA.2, suggesting that BA.2 is more tightly bound than the others, consistent with the smaller $d_{\text{hACE}2}^{\text{RBD}-A}$ of BA.2. Fig. 4 shows selected contacts of BA.2 from RL. More details can be found in the PDB files as part of the supporting material.

**Figure 5:**
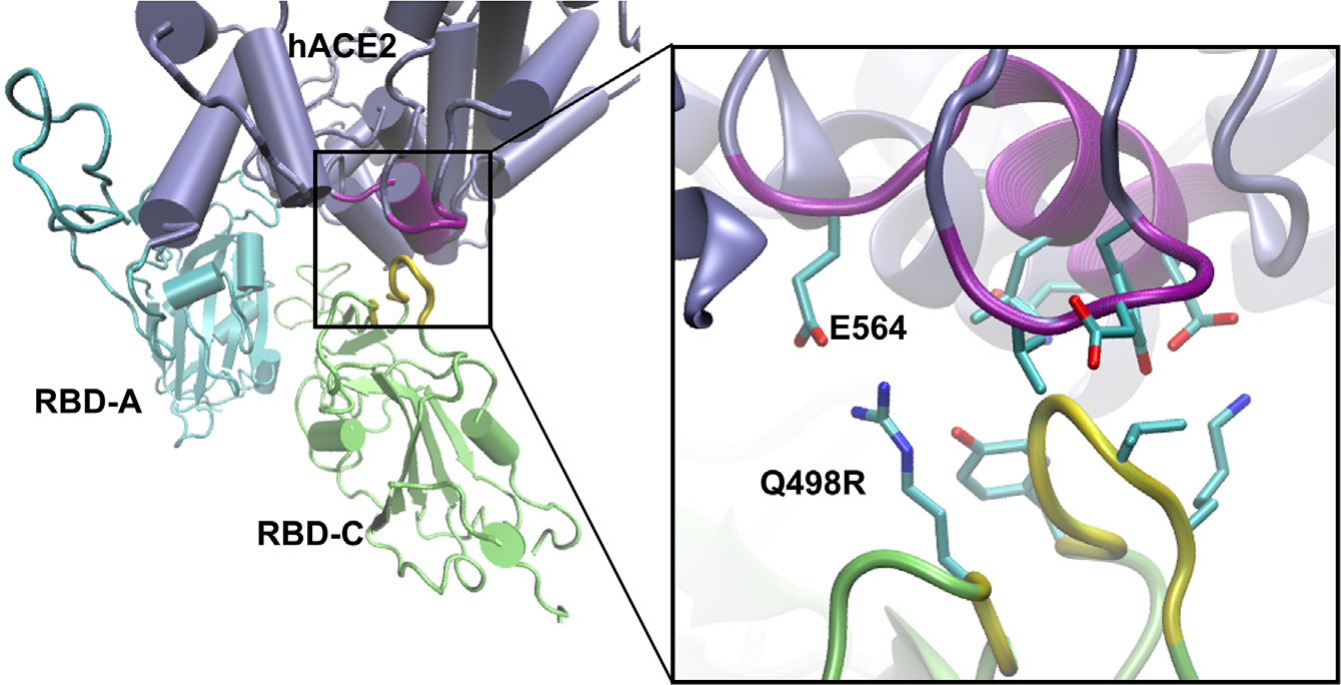
BA.2 (RL): interfacial charged contact E564_hACE2_···Q498R_C_.

Up to this point, we have shown that RL and RS share similar range for COM distance among the variants. For the interface between RBD-A and hACE2, both replicas show multiple RBD-A···hACE2 contact partners with different occurrence probabilities, implying that the interfacing is dynamic. Of great interest and yet to be understood is why BA.2 of RL has a 5 Å shorter $d_{\text{hACE}2}^{\text{RBD}-C}$ than RS. Next, we analyze the interactions at the interface between RBD-C and hACE2 of RL, and then show how they differ from the RS replica. In brief, we find that RBD-C of BA.2 forms contacts to hACE2 for RL. Based on the much shorter $d_{\text{hACE}2}^{\text{RBD}-C}$ for BA.2, we hypothesized that its RBD-C can interact with hACE2, and carried out a similar contact probability analysis (as done for RBD-A) to see whether any contacts between RBD-C and hACE2 exist. We find that there are no close contacts formed for WT or BA.1, while a substantial amount of interaction is found for BA.2 ( >99% for residues 445–449 and 498; 30–40% for residues 483–484, see Fig. S9). These contacts were not initially present, but were formed during equilibration of the simulation. Table 3 shows the major contact partners between RBD-C and hACE2, which are also depicted in Figs. 5 and S9. These hACE2 residues are different from those that bind to RBD-A (Table 2), and therefore contribute additional binding between hACE2 and S-protein. Yin et al. (46) observed that a monomeric hACE2 binds to a trimeric Omicron S-protein with sixfold higher binding affinity than to WT S-protein, and only twofold higher affinity when binding to an Omicron monomer S-protein than to WT. From which we can infer that, for Omicron, more than one RBD can bind onto one monomeric hACE2 as seen in RL of BA.2.

**Table 3:** Contact residues between RBD-C of BA.2 and hACE2

RBD-C_BA.2_···hACE2
V445/G446···V212
G446···D213
K444/V445/G446···Y215
Q498R···E564
G446/Y449···L568
K444···E571
Y449···N572

### RBDs of A and C chain are decoupled in BA.2

**Figure 6:**
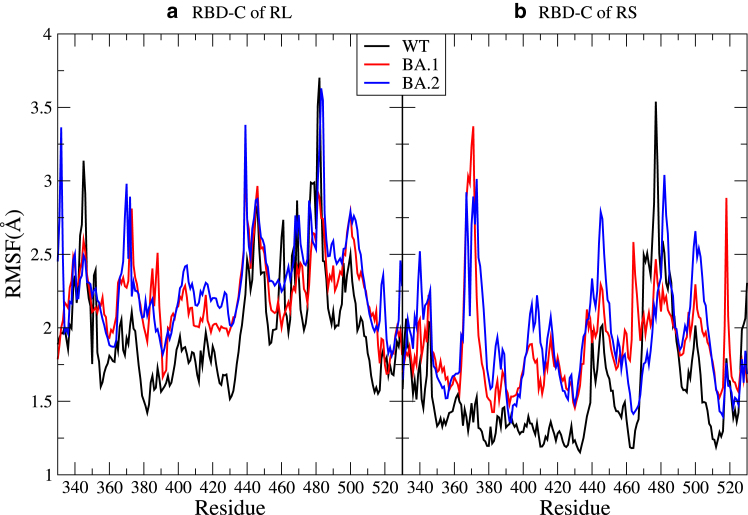
RMSF profiles for RBD-C of S-protein: RL (*a*) and RS (*b*): RBD-C of the Omicrons has higher RMSF than that of WT.

In Fig. 6 we show the root mean-square fluctuation (RMSF) of the RBD-C residues. The RMSF of all RBDs can be found in Fig. S10. We find that the RBD-C of the Omicrons, in particular BA.2, have higher RMSF than WT: the RMSF average values over RBD residues for WT/BA.1/BA.2 in Å are 2.01/2.22/2.33 (RL) and 1.57/1.87/1.92 (RS), implying an increased flexibility in RBD-C of the Omicrons.

From this we hypothesized that RBD-C’s ability to form contacts with h-ACE2 has to do with its increased freedom to restructure due to having less contact with the other RBDs. We hence investigated the degree of interaction between the RBDs and searched for the key differences invoked by the mutations. Figs. S11 *b* and *c* and S12 *b* and *c* show the probability change in the interdomain RBD-A···RBD-C contact from WT to BA.1/BA.2 (the full contact probabilities are shown in Figs. S11 *a* and S12 *a*) and we find that the total probability of contact remains approximately unchanged between WT and BA.1, whereas a clear reduction is seen for BA.2. Which demonstrates that there is a decoupling of the RBD-A and RBD-C in BA.2.

This is due to several differences in RBD-A···RBD-C contact between the variants, over the regions 400–420_C_ and 440–500_C_. The latter is within the receptor binding motif of C chain (RBM_C_) and is also exposed to hACE2. For region 400–420_C_ there is an enhancement of interactions for BA.1 (RL) but loss of interactions for BA.2 (both RL and RS). In none of our models are there any contacts with hACE2 within this region. Here, BA.1 of RL exhibits an additional contact made with R408_C_, which is not present in either BA.2 or WT. Furthermore, for BA.1, R408_B_ forms contacts with residues 374 to 377 on RBD-C (nearly 100% for both RL and RS). These contacts are missing in both BA.2, which has the mutation of R408S, and WT. We note that R408_C_ is located in the inner cavity formed by the three RBDs. D428_A_ and R408_C_ of BA.1 (RL) form a salt bridge (while in RS, the two residues are associated via both being attracted to a glycan $\bar{N}$234_RBD-B_). This contact to D428_A_ is largely absent for BA.2 because of the R408S mutation. For WT, D428_A_ connects to Y505_C_ highlighting the different relative position of RBD-C compared with the Omicrons.

**Figure 7:**
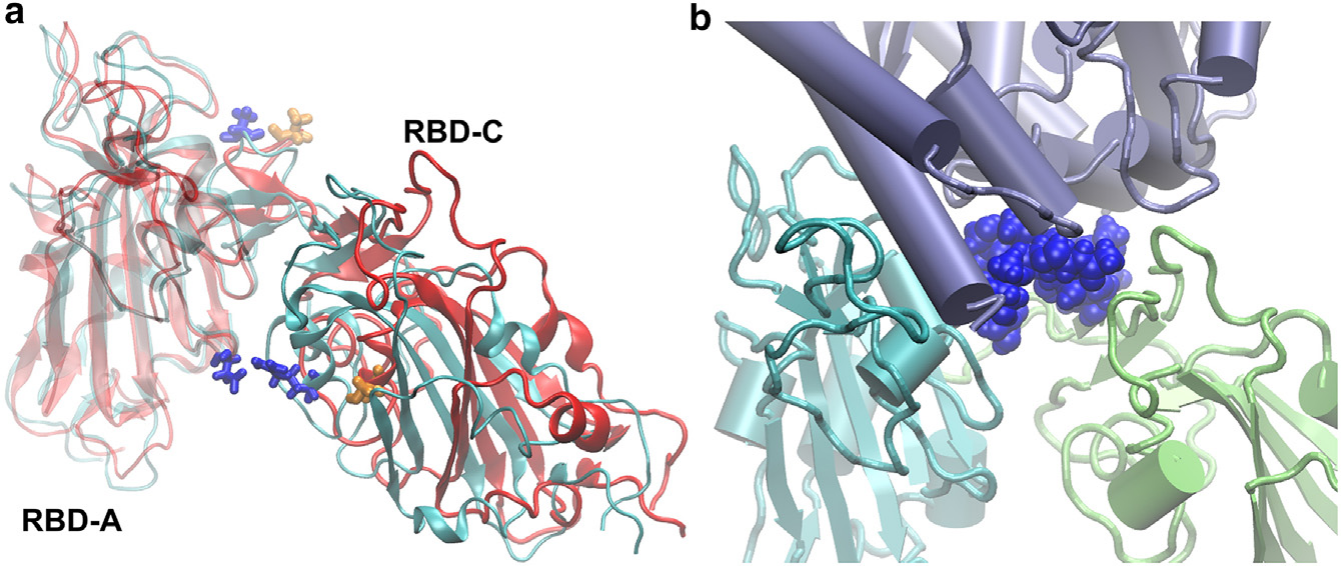
(*a*) Relative positions of RBD-A and RBD-C of BA.1 (*cyan*) and BA.2 (*red*). Aligned with RBD-A, it can be seen that RBD-C of BA.2 is further away from its RBD-A than BA.1. In the foreground are D428_A_···R408_C_ (*blue*) for BA.1, and residues R408S_C_ of BA.2 (*orange*). In the background are E484A_C_ of BA.1 (*blue*) and BA.2 (*orange*). (*b*) Viewing from the inner cavity side, RBD-A and RBD-C are so decoupled that $\bar{N}$90_hACE2_ (*blue spheres*) can even occupy the gap in between. (Corresponding figures for RS are shown in Fig. S13).

Regarding RL, the reduction in RBD-A···RBD-C interactions after mutation—over the region 440–500_C_, i.e., RBM_C_—corresponds to the increase in interaction RBD-C···hACE2, as seen earlier, suggesting that RBD-C is pulled away from interchain S contact toward hACE2. By inspecting the solvent-exposed side of RBD-A and RBD-C of WT, we find that a salt bridge, K378_A_···E484_C_, is the dominant interaction, and to a lesser extent, a hydrogen bonding pair, S375_A_···E484_C_, forms at this interface. Mutation E484A results in the loss of this salt bridge (shown in the probability difference in Fig. S11, *b* and *c*) and gives further flexibility to RBD-C for both BA.1 and BA.2. Thus, E484A loosens one connectivity between RBD-A and RBD-C for both BA.1 and BA.2, whereas the R408S mutation on RBD-C of BA.2 further loosens its connections to both RBD-A and RBD-B, thereby setting BA.2 apart from BA.1, and gives BA.2 the most flexible RBD-C. Fig. 7 *a* depicts the contact pair D428_A_···R408_C_ of BA.1, and shows that E484A_C_ of the Omicrons are not in close contact to RBD-A. The extent of decoupling between RBD-A and RBD-C can be seen in Fig. 7 *b*.

### Interaction mediated by glycans

The final piece of the puzzle of how hACE2 interfaces with S-protein lies in the role that glycans play in the interaction. Of particular interest are $\bar{N}$90_hACE2_, $\bar{N}$322_hACE2_, and $\bar{N}$165_NTD-A_, located near the interface between hACE2 and S-protein, of which $\bar{N}$90_hACE2_ and $\bar{N}$322_hACE2_ are predicted to interact with S-protein (26,34). $\bar{N}$165 on NTD-B was suggested to have a stabilizing role for RBD-A at the up conformation by Casalino et al. (47). Here, we find that $\bar{N}$165_NTD-A_ and $\bar{N}$90_hACE2_ have an involved role in the RBD-C···hACE2 interface.

The interaction between $\bar{N}$322_hACE2_ and a monomeric S-protein was shown to be transient in a simulation study by Nguyen et al. (48). Shajahan et al. (34) also pointed out that the site N322 is aligned in human, bat, cat, pig, and chicken, but that pig and chicken are not susceptible to SARS-CoV-2. By visual inspection we find that $\bar{N}$322_hACE2_ is exposed to the solvent. Indeed, we do not find substantial contact between $\bar{N}$322_hACE2_ and the RBDs, which is in line with the earlier findings.

In contrast, animal species that are susceptible to SARS-CoV-2 have an N90 site while nonsusceptible species do not (34), and $\bar{N}$90_hACE2_ was shown to contact RBD with high probability (48), suggesting a modulating role of $\bar{N}$90_hACE2_. Mehdipour and Hummer (26) showed that $\bar{N}$90_hACE2_ interferes with the binding between hACE2 and S-protein, whereas, Chan et al. (49) designed a decoy soluble hACE2, mutated at N90 to remove its N-glycan and obtained high binding affinity to S-protein.

Our results on WT show that, $\bar{N}$90_hACE2_ interacts with RBDs mostly on N460_A_ and T500_C_, both facing the inner cavity of the RBDs, and that $\bar{N}$90_hACE2_ drops orthogonal to the S+hACE2 interface resulting in a comparatively large space between hACE2 and S-protein, reflected in its large COM distance $d_{\text{hACE}2}^{\text{RBD}-C}$ (Table 1). The scenario of $\bar{N}$90_hACE2_ giving a gap between RBDs and hACE2, is consistent with the experimental observation of Mehdipour and Hummer (26) on its interfering role in binding.

On the other hand, all replicas for the Omicrons share similar behavior for $\bar{N}$90_hACE2_—i.e., it enters the RBD–hACE2 interface (Figs. S14, S17, and S18). Observing them reveals that Q498R_C_ plays the key role in attracting and leading $\bar{N}$90_hACE2_ into the interface to further interact with the many hydrophilic sidechains within (see the video as part of the supporting material). Substantial contacts form between $\bar{N}$90_hACE2_ and RBD-A of BA.1/BA.2—involving residues 403 to 409, and T415—and spread extensively over RBM_C_, e.g., Y449, Q493R, Q498R, and N501Y, which directs $\bar{N}$90_hACE2_ toward the center of the interface. Compared with BA.2, BA.1 has fewer contacts between RBD-C and $\bar{N}$90_hACE2_, presumably due to the above-mentioned higher degree of RBD-A···RBD-C interaction resulting in its lower RBD-A/RBD-C flexibility. The contacts of $\bar{N}$90_hACE2_ on RBM_C_ bring RBD-C to the vicinity of hACE2 reducing the interfacial gap, as reflected by the smaller $d_{\text{hACE}2}^{\text{RBD}-C}$, and align RBM_C_ to the interface of S+hACE2. In Omicrons, $\bar{N}$90_hACE2_ is orientated parallel to the interface between RBD and hACE2: we measured the orientation angle ($\Theta_{N90}$) of $\bar{N}$90_hACE2_ with respect to the interface (see Fig. S15) and found that $\bar{N}$90_hACE2_ is close to parallel to the S+hACE2 interface in the case of BA.2 (average $\Theta_{N90}$ = 9°/26°) and BA.1 ($\Theta_{N90}$ = 20°/9°), but more orthogonal for WT ($\Theta_{N90}$ = 74°/92°) with values given for RL/RS, respectively. The unrestrained replica of BA.2 also has a low average $\Theta_{N90}$ = 21°. Therefore, for the Omicrons, $\bar{N}$90_hACE2_ no longer acts as a spacer between RBD and hACE2, but as a glue in between.

In addition, we find $\bar{N}$165_NTD-A_ at this interface: it is found alone for the WT, whereas it shares the interface with $\bar{N}$90_hACE2_ for BA.1, and for BA.2 $\bar{N}$165_NTD-A_ seems more mobile, likely driven by the mutations of D405N and R408S on RBD-A that weaken the connection of $\bar{N}$165_NTD-A_ to the center of the interface. All BA.2 replicas show $\bar{N}$165_NTD-A_ distancing itself from the interface center, e.g., in replica RL $\bar{N}$165_NTD-A_ is out of the interface (Fig. S14).

**Figure 8:**
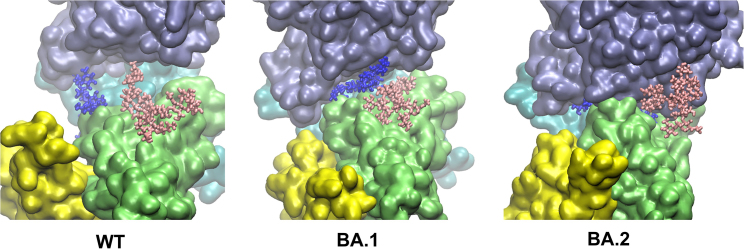
Positions of glycans $\bar{N}$90_hACE2_ (*blue*) and $\bar{N}$165_NTD-A_ (*pink*) relative to hACE2 (*lilac*) and RBDs—RBD-A (*cyan*), RBD-B (*yellow*), RBD-C (*lime*)—at the end of 1 *μ*s. The interface is widest for the WT, and narrowest for BA.2.

Our simulations thus suggest that glycans take part in interfacing Omicrons with hACE2. Fig. 8 shows the positioning of $\bar{N}$90_hACE2_ and $\bar{N}$165_NTD-A_ between hACE2 and RBDs of WT, BA.1, and BA.2. These two glycans modulate the spacing of the interface. Depending on the orientation of the more branched $\bar{N}$165_NTD-A_, the interfacial width can be widened as in the BA.1 RL replica, where it stacks on $\bar{N}$90_hACE2_, and when it leaves the interface as in the case of the BA.2 RL replica, the space becomes so narrow that RBD-C can bind to hACE2.

For both BA.1 and BA.2, the presence of the two glycans—$\bar{N}$90_hACE2_ and $\bar{N}$165_NTD-A_—at the interface mediate the interactions between RBD and hACE2. This is supported by what was found by Huang et al. (50), who experimentally showed that the glycan interaction made the S···hACE2 contact stronger. Using atomic force microscope te Riet et al. (51) showed that the strengthening of pathogen-receptor binding was mediated by glycan-protein lateral interactions. It is conceivable that the Omicrons adopt this strategy to become more infectious.

## Conclusion

In this work, we used MD simulations to investigate the SARS-CoV-2 S+hACE2 structures, with S-protein from WT, and the Omicron subvariants BA.1 and BA.2. Starting from the 1-up conformation given by Woo et al. (29), we find two types of interfacing between S-protein and hACE2. One involving predominately the up chain A (seen for WT and being of the same form as the starting structure) and a more compact conformation involving both chains A and C (seen for the Omicrons). For the former, while the mutation on RBD increases the electrostatic attraction toward hACE2, we observe swaps in the binding partners in the neighborhood with different probabilities. For the latter, the involvement of chain C is through the injection of the glycan $\bar{N}$90_hACE2_ between hACE2 and S-protein driven by Q498R_C_. This gives rise to the glycan-protein lateral interactions, namely, RBD-C···$\bar{N}$90_hACE2_···hACE2. In addition, glycan $\bar{N}$165_NTD-A_ also influences the interfacial gap by staying or leaving the interface. When it leaves, seen in the RL of BA.2, RBD-C···hACE2 is formed. We observe the glycan-protein interplay at the interface of RBD-C and hACE2 in all Omicron replicas, including the unrestrained BA.2 replica. The factors that promote this interacting mode are as follows: 1) Q498R_C_ on the Omicrons attracts $\bar{N}$90_hACE2_ into the interface, where RBD-C forms a broad set of contacts with the $\bar{N}$90_hACE2_ spreading across the interface between RBD-C and hACE2 (see Figs. 8 and S14), resulting in a narrower interfacial gap and 2) there is less coupling between RBD chains in Omicrons, in particular BA.2, than WT which makes them more flexible and able to approach hACE2.

The mutation of E484A in BA.1/BA.2 abolishes a salt bridge K378_A_···E484_C_ (at the solvent-facing side) present in WT. The mutation of R408S in BA.2 abolishes another salt bridge D428_A_···R408_C_ (facing toward the inner cavity) found in BA.1, as well as cutting the connection with RBD-B (e.g., R408_B_···F374_C_ in BA.1), and therefore further reducing the connectivity to RBD-C. The mutations E484A, R408S, and D405N in our result affect the structure, and the latter two make BA.2 distinct from others in the study. These mutations are maintained in the later, and more infectious, subvariants such as BA.4 (52), BA.5 (53), and Arcturus (54), suggesting that these are indeed favorable mutations for increasing the fitness of the virus. Moreover, the high degree of interaction between glycans on hACE2 and S-protein indicates that it is advantageous to consider such glycan-related epitopes in the therapeutic design. It has been suggested that the increased electrostatic attraction between S-protein and hACE2 may simply enhance the binding. Here, we find that it leads also to additional binding involving glycan lateral interactions or multiple RBDs with the hACE2 monomer.

The structural measures introduced and investigated here can be more generally used to monitor emerging variants and future evolution of the virus thereby enabling better models of the biophysical basis for evolutionary advantage.

## Author contributions

D.J.B. and J.C. obtained the funding. Y.-W.H., D.J.B., and J.C. designed the research. Y.-W.H. carried out simulations and data analysis. Y.-W.H., T.T., and G.J.S. contributed analytic tools. All authors contributed to the writing of the article.

## Supporting material

Document S1. Figures S1–S18 and Tables S1–S3

Data S1. PDB of the final structure BA.1 for RL

Data S2. PDB of the final structure BA.2 for RL

Data S3. Video of glycan N90 _hACE2_ entering the interface between S of BA.2 and hACE2 for RL

Data S4. PDB of the final structure WT for RL

Document S2. Article plus supporting material
