# Supplementary material for: Structure adaptation in Omicron SARS-CoV-2/hACE2: Biophysical origins of evolutionary driving forces
Source: Biophys J. 2023 Sep 16;122(20):4057–67. doi: 10.1016/j.bpj.2023.09.003 (PMC10624932; doi:10.1016/j.bpj.2023.09.003)
Supplement: Document S1. Figures S1–S18 and Tables S1–S3 [file mmc1.pdf]

Biophysical Journal, Volume *122*

Supplemental information

Structure adaptation in Omicron SARS-CoV-2/hACE2: Biophysical origins of evolutionary driving forces

Ya-Wen Hsiao, David J. Bray, Tseden Taddese, Guadalupe Jiménez-Serratos, and Jason Crain

Key Measurements Made

Trajectory data from the production run was obtained by taking snapshots of the system every 20 ps. As explained in the results the initial simulation structure equilibrates over the first part of the simulation. We therefore analysed the data from the last 400 ns changesand 100 ns for replicas 1 and 2, respectively, of the simulation.

To study the interaction between amino acids we used the concept of close contact whereby a pair of residues was said to be in contact if the minimum distance between inter-residue atoms was less than 3 Å. We note that by doing so, we capture potential van-der-Waals, hydrogen bond and ionic attractions. We also refer to these close contacts as having direct interaction.

The probability of occurrence of a close contact involving the residue was given as the number of frames in which the contact occurred divided by the total number of frames evaluated. Only events with probability higher than 50% are reported, unless otherwise noted.

Similarly, the centre of mass (COM) of a chain domain is defined as the non mass-weighted average position using every atom of the domain. The distance between domains α and β, $d^{\text{ff}}_{\text{fi}}$, is then calculated as the length between their COM.

The RBD opening angle of chain A (as used by Fallon et al. (42)), $\theta_{\text{RBD-A}}$, is defined between the COMs of residues 338-517 of chain A, 324-327 and 538-585 of chain A, and 747-755 of chain C. By rotational symmetry, $\theta_{\text{RBD-C}}$ is defined the same as above but replacing A$\rightarrow$C and C$\rightarrow$B.

Root mean square fluctuation (RMSF) was calculated using VMD command *measure rmsf*, by comparing Cα coordinates to the reference structure which is the time average over 600 ns to 1 μs (replica RL), and 400 to 500 ns (replica RS).

When studying glycans, the orientation of $\bar{\text{N}}90_{\text{hACE2}}$ with respect to S+hACE2 interface, was quantified by measuring the angle, Θ_{N90}, made between the long axis defined by atom C1 on residues 1 to 6 of $\bar{\text{N}}90_{\text{hACE2}}$ and the axis defined by residues 547 and 560 of hACE2 (see right panel of Figure S15).

Additional information

"movie:BA2-init.gif" First 40 ns of BA.2 from the 1 µs simulation. Q498R_{C} catches $\bar{\text{N}}90_{\text{hACE2}}$ (blue) to enter the interface and $\bar{\text{N}}165_{\text{NTD}-\text{A}}$ (pink) leaves the interface. Tip of $\bar{\text{N}}90_{\text{hACE2}}$ is in lighter blue.

Table S1. Key segment names in the PDBs

	WT	BA.1	BA.2
spike chain A	PROA	PROA	PROA
spike chain B	PROB	PROB	PROB
spike chain C	PROC	PROC	PROC
hACE2	PROD	PROD	PROD
$\bar{N}90_{hACE2}$	CABI	CABK	CABK
$\bar{N}322_{hACE2}$	CABL	CABL	CABM
$\bar{N}165_{NTD-A}$	CARF	CAAT	CAAT

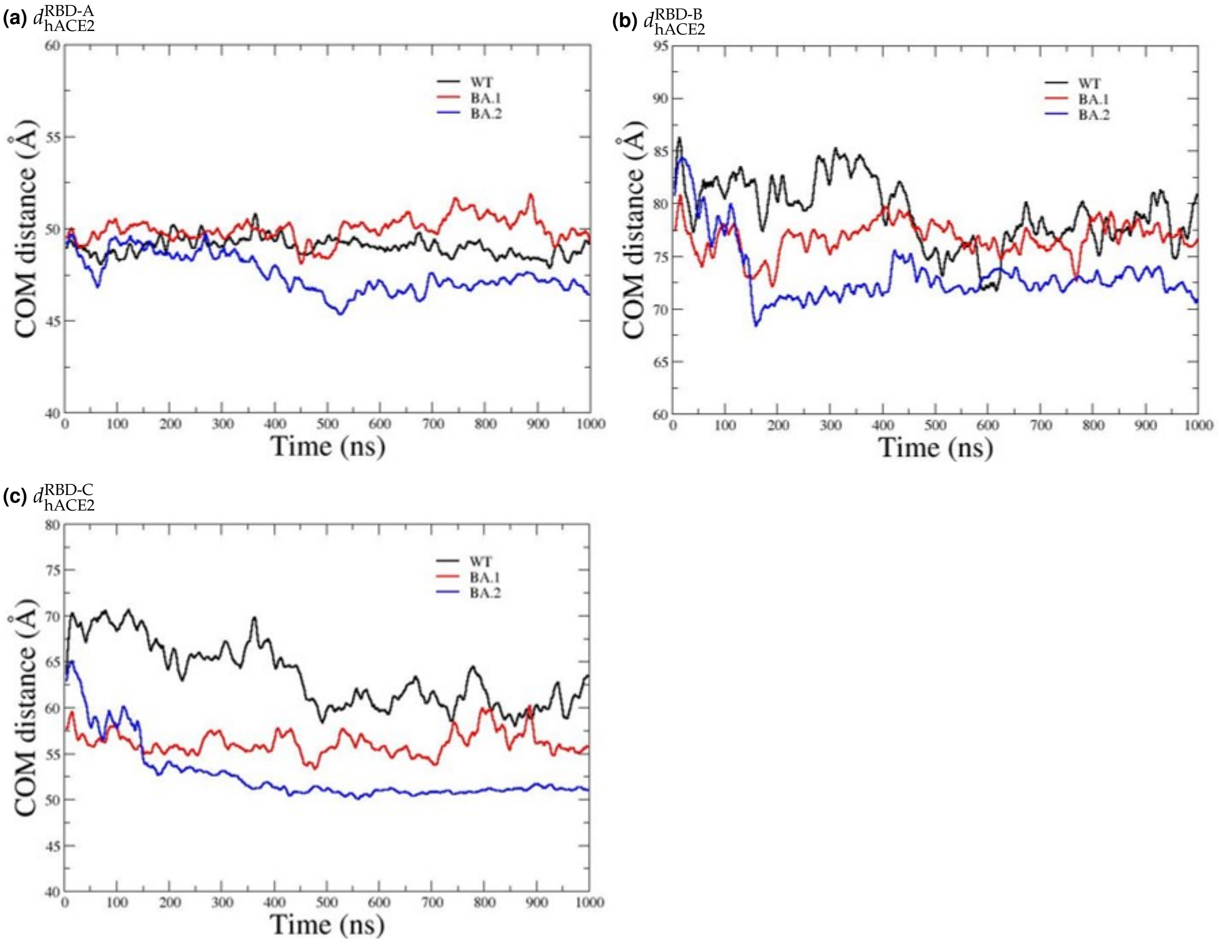

Fig. S1. Centre of mass distances between hACE2 and the RBD of chains A, B, and C of replica RL, respectively

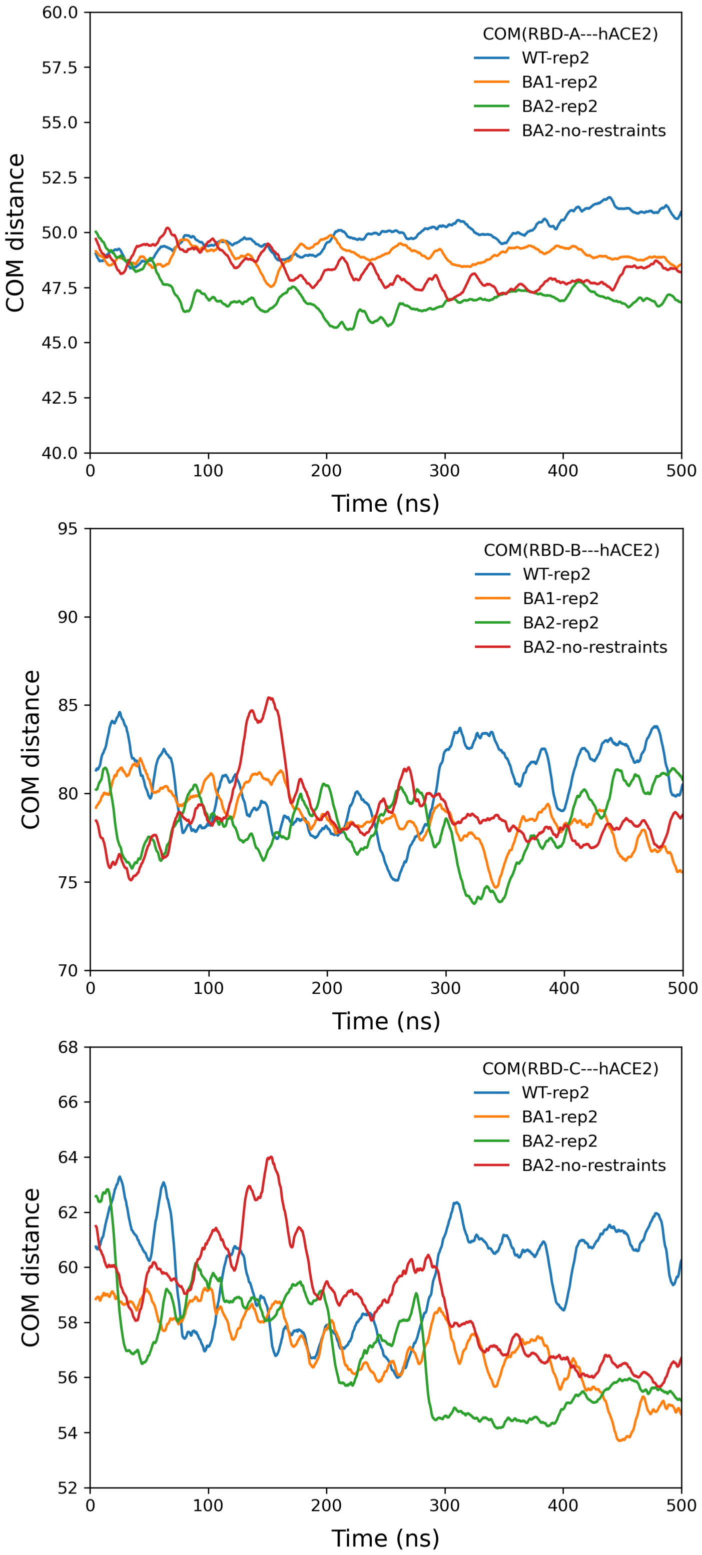

Fig. S2. Centre of mass distance between hACE2 and RBD of chains A (top), B (middle), and C (bottom) for replica RS

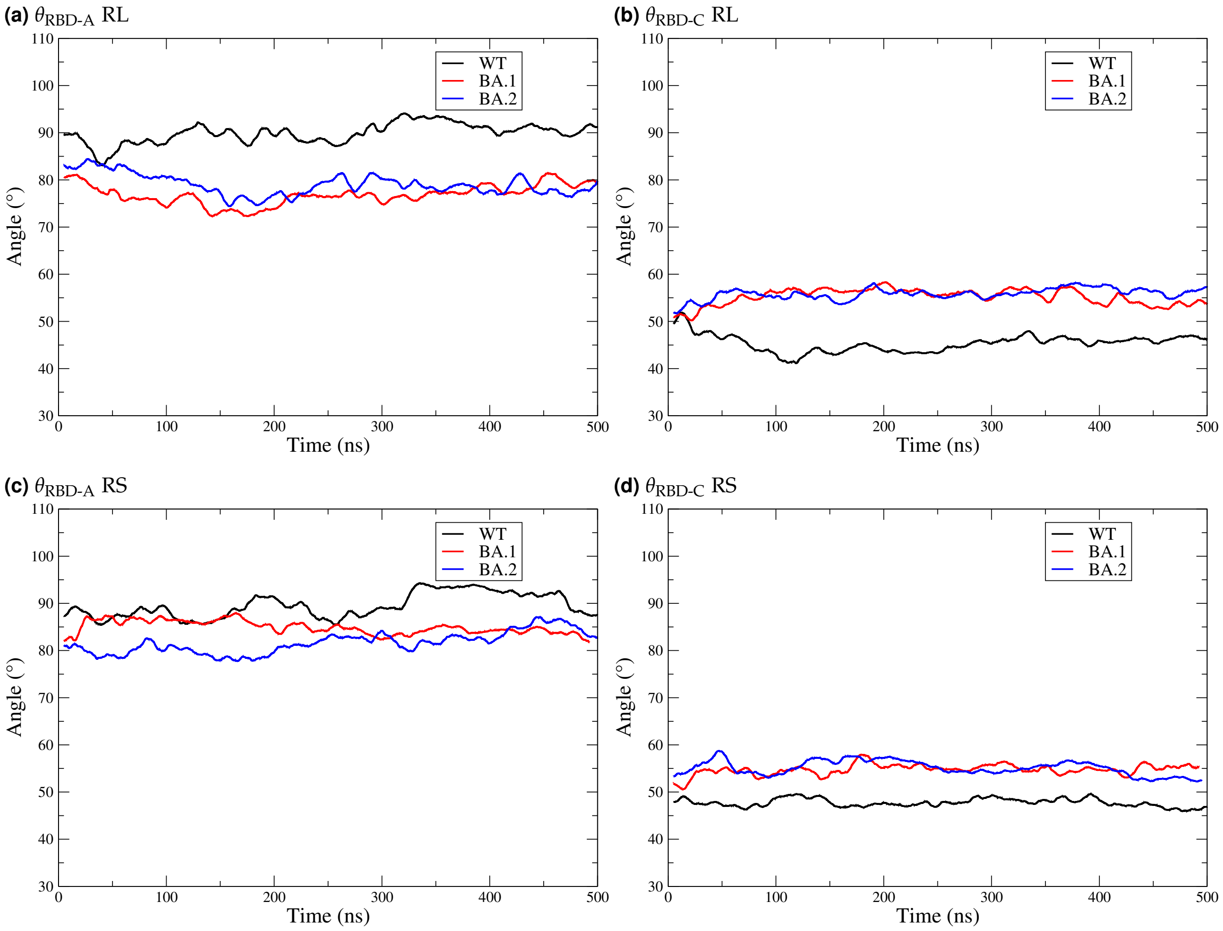

Fig. S3. Opening angle measurement of the up-chain (chain A: (a), (c)) and its clockwise neighbor chain (chain C: (b), (d)) of the S-protein: data are 10-ns running averaged

Table S2. Replica BA.2 without restraints: Most probable contact pairing between key mutated residues of RBD-A and hACE2 with % occurrence given in brackets. Entries in bold indicate contact involving the mutated residue.

Residue	BA.2$_{no-restraints}$
K417**N**	–
N440**K**	**E329 (19%)**
S477**N**	S19 (25%)
T478**K**	–
E484**A**	K31 (3%)
Q493**R**	**D38 (99%)/E35 (56%)/H34 (97%)/K353 (46%)**
G496**S**	–
Q498**R**	**Y41 (59%)/Q42 (42%)/L45 (15%)**
N501**Y**	**K353 (100%)/D355 (77%)/Y41 (56%)**
Y505**H**	**K353 (95%)/R393 (71%)/G354 (63%)/A387 (49%)/E37 (53%)/A386 (76%)**
D405**N**	**A387 (77%)**
R408**S**	–

Table S3. Breakdown list of contact pairs involving key mutated residues of RBD-A found across replicas with % occurrence given (in order of RL, RS and unrestrained replica).

RBD-A···hACE2	WT	BA.1	BA.2
D405**N**···A387	3 ,—	—,—	71 , 36, 77
K417**N**···D30	90 , 47	—,—	—,—,—
N440**K**···E329	—,—	27 ,—	90 , 74, 19
S477**N**···S19	—,—	—, 75	13 ,—, 25
E484**A**···K31	17 , 93	—,—	—,—,—
Q493**R**···K31	60 , 43	—,—	—,—,—
Q493**R**···H34	—, 50	100, 93	99 , 86, 97
Q493**R**···E35	77 , 46	—, 97	—,—,—
Q493**R**···D38	—,—	100, 35	99 , 93, 99
Q493**R**···K353	—,—	95 ,—	77 , 14, 46
Q493**R**···D38	—,—	—,65	—,—,—
G496**S**···K353	23 ,—	77 , 67	—,—,—
Q498**R**···D38	—,—	—, 99	—,—,—
Q498**R**···Y41	81 , 1	94 , 86	90 ,84, 59
Q498**R**···Q42	41 ,—	42 , 84	37 ,63, 42
N501**Y**···Y41	86 , 68	77 , 76	30 , 72, 56
N501**Y**···K353	100, 82	100, 100	100, 99,100
N501**Y**···D355	100, 18	100, 23	100, 84, 77
Y505**H**···E37	100,—	—,—	—, 23, 53
Y505**H**···K353	100, 95	98 , 99	63 , 95, 95
Y505**H**···A386	—,—	—,—	98 , 19, 76
Y505**H**···A387	—,—	—,—	98 , 59, 49
Y505**H**···R393	99 ,—	—,—	—,—, 71

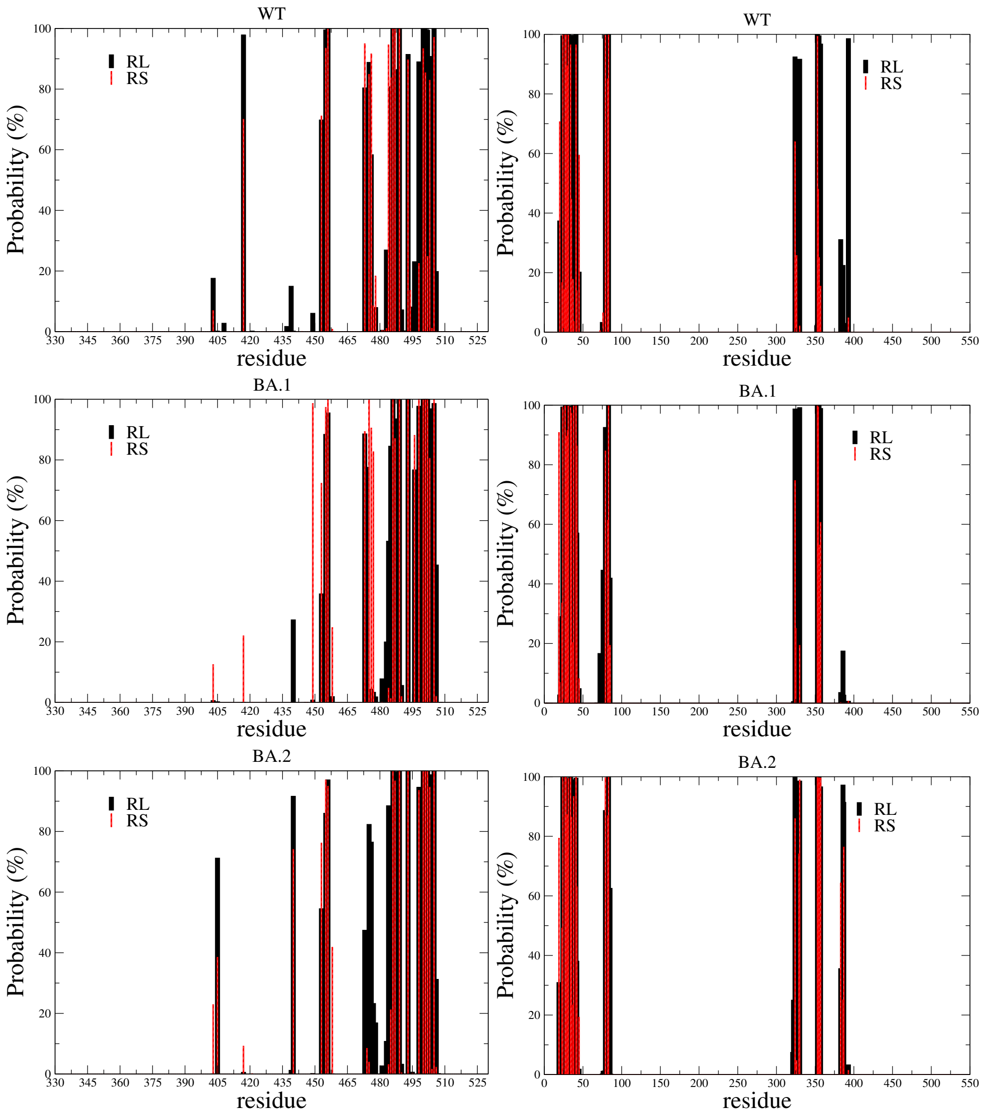

Fig. S4. Left column: Probability of the residues on RBD-A within 3 Å of hACE2; right column: Probability of the residues on hACE2 within 3 Å of RBD-A

(a) BA.1 to WT

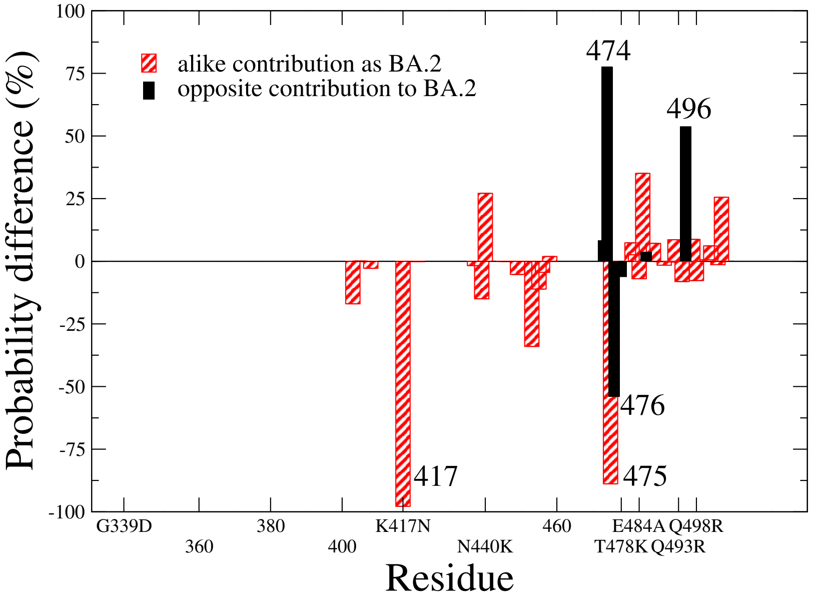

(b) BA.2 to WT

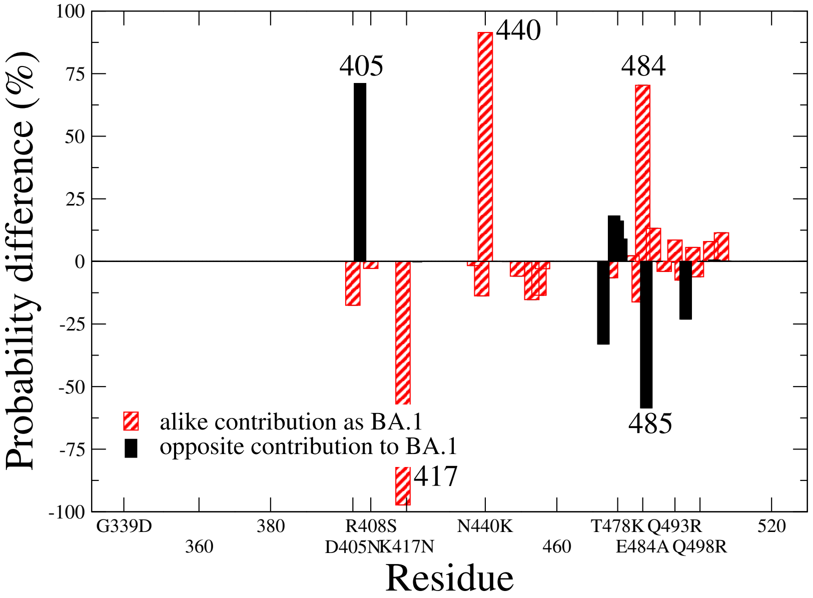

Fig. S5. Probability difference of RBD-A residues of S-protein in contact with hACE2 ((a), (b)). Residues contributing more than 50% are labelled. (replica RL)

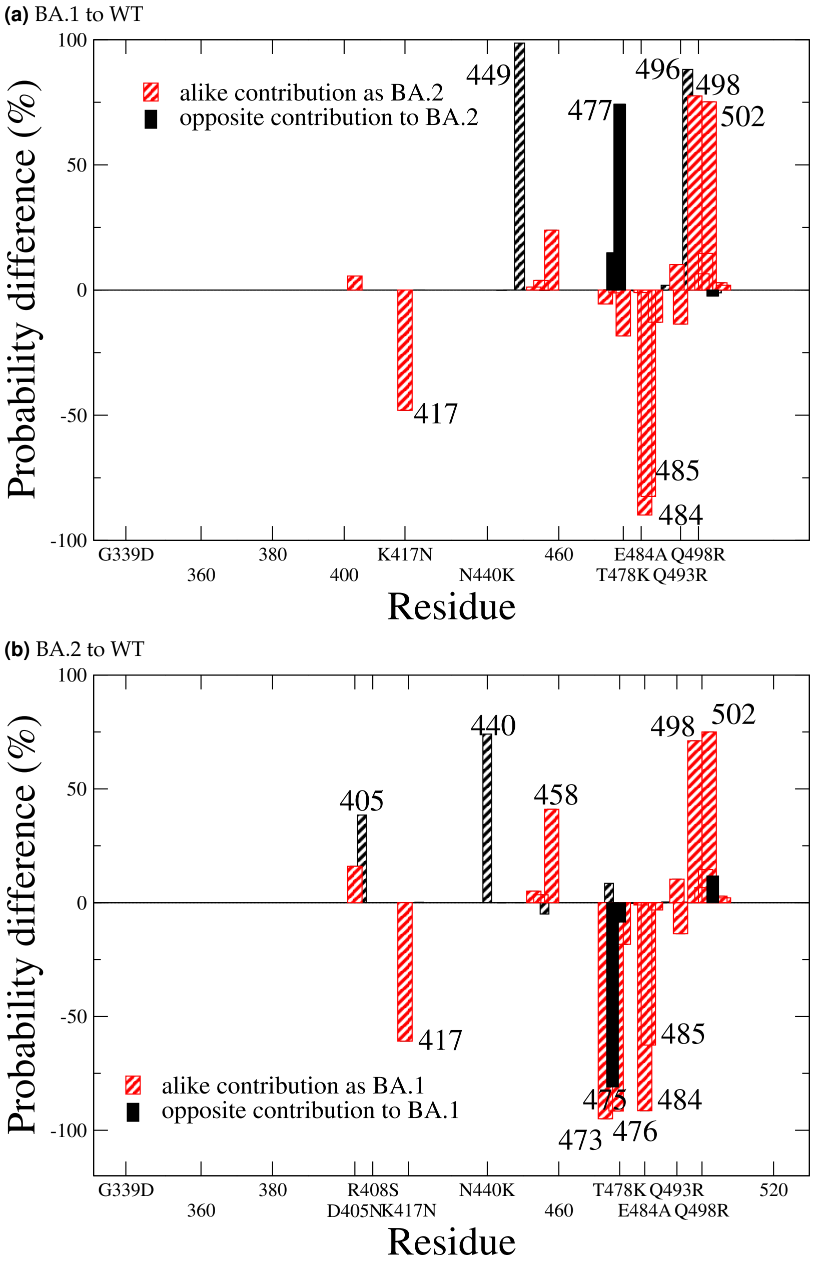

Fig. S6. Probability difference of RBD-A residues of S-protein in contact with hACE2 ((a), (b)). Residues contributing more than 50% are labelled. (replica RS)

(a) BA.1 to WT

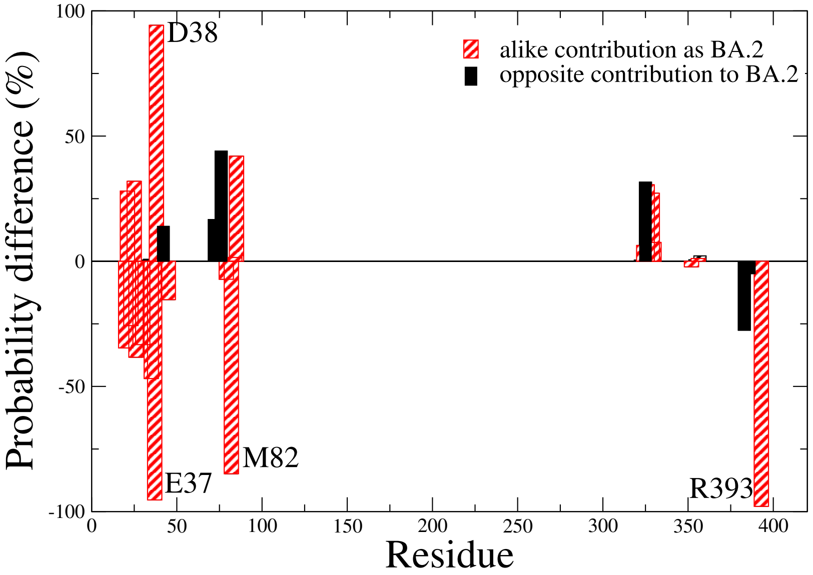

(b) BA.2 to WT

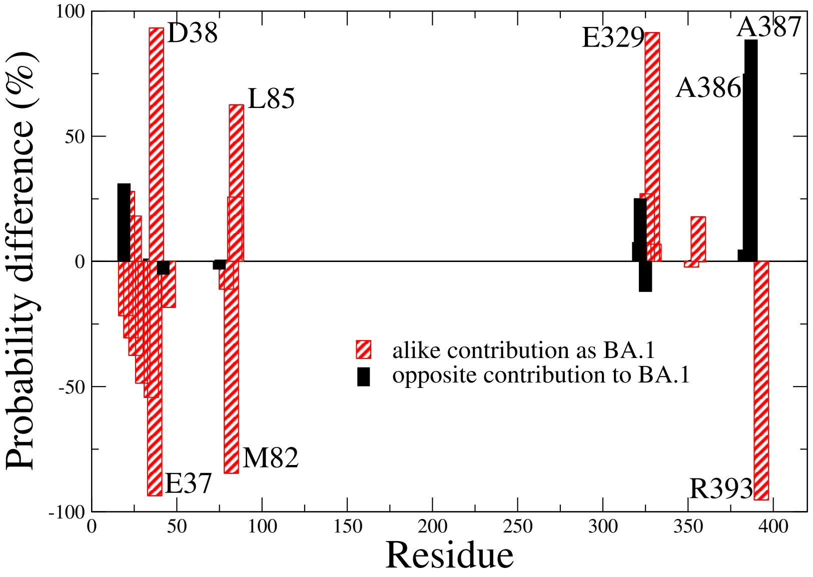

Fig. S7. Probability difference of hACE2 residues in contact with RBD-A of S-protein ((a), (b)). Residues contributing more than 50% are labelled. (replica RL)

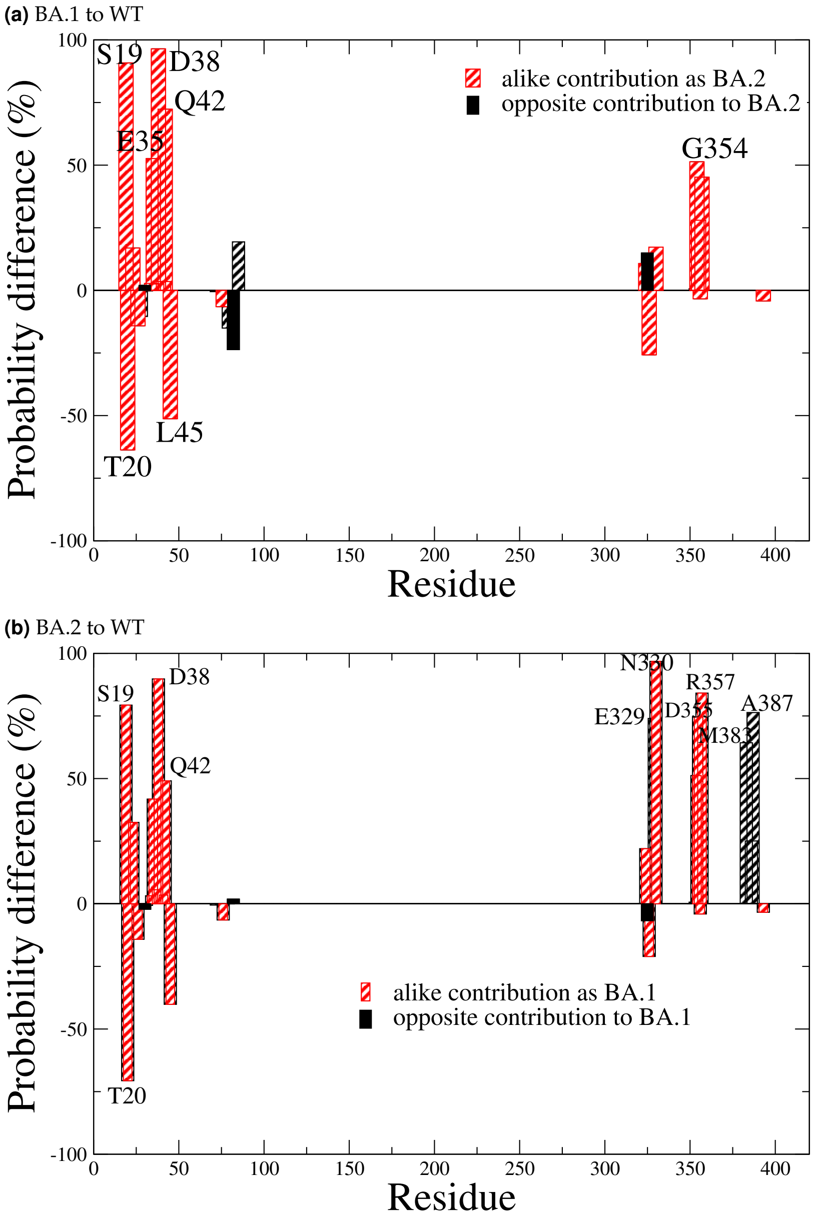

Fig. S8. Probability difference of hACE2 residues in contact with RBD-A of S-protein ((a), (b)). Residues contributing more than 50% are labelled. (replica RS)

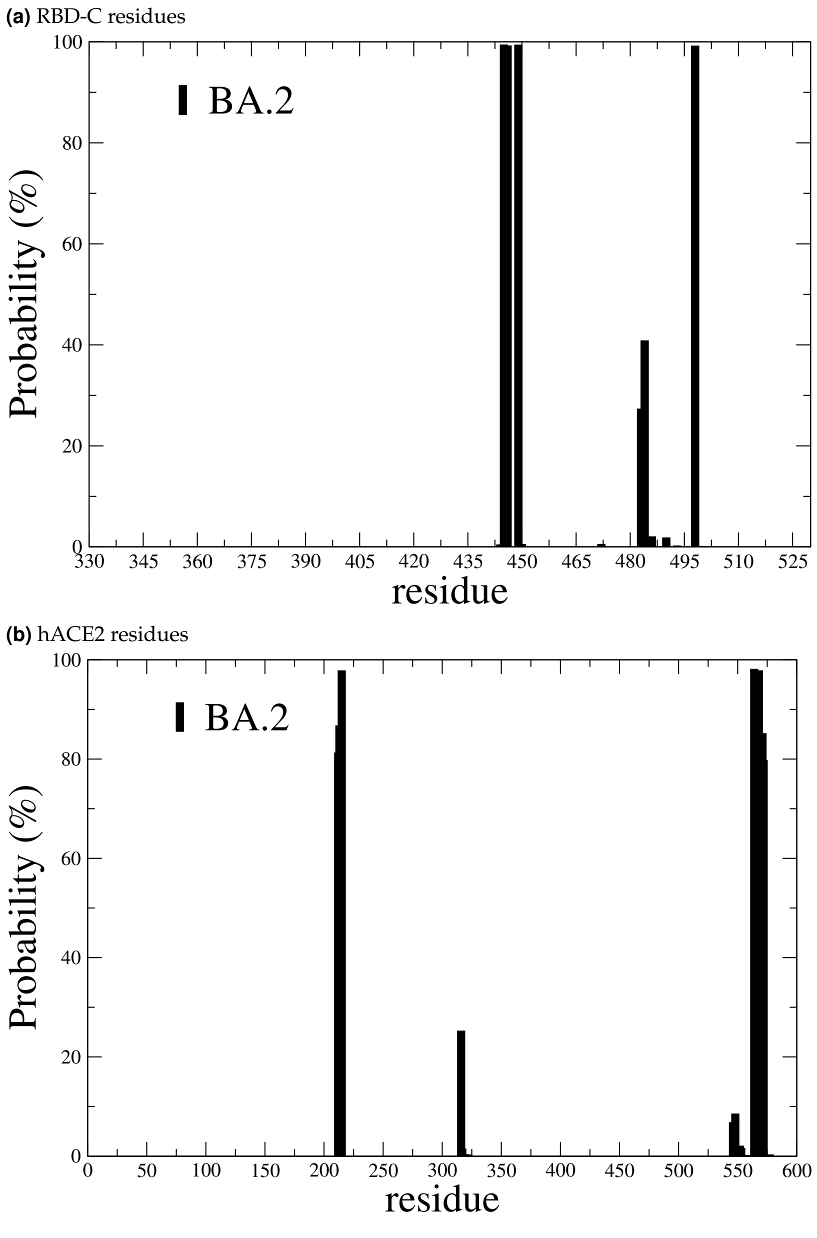

Fig. S9. From RL: Probability of BA.2 RBD-C in contact with hACE2: residues on RBD-C (a); residues on hACE2 (b)

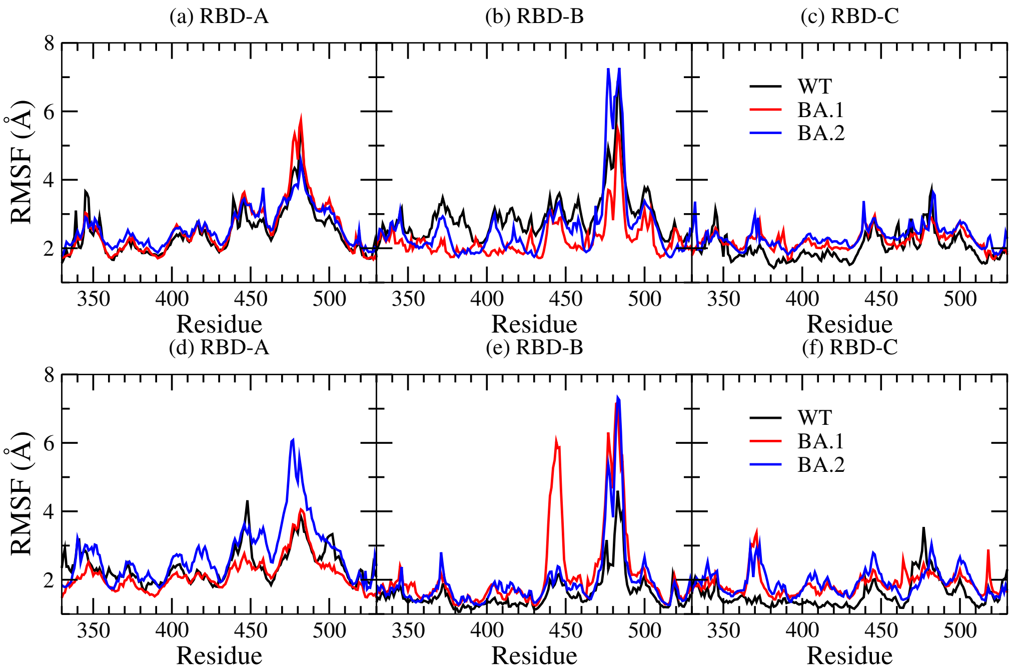

Fig. S10. RMSF profiles for RBDs of S-protein: RL (a-c) and RS (d-f)

(a) Probability of residue contacts

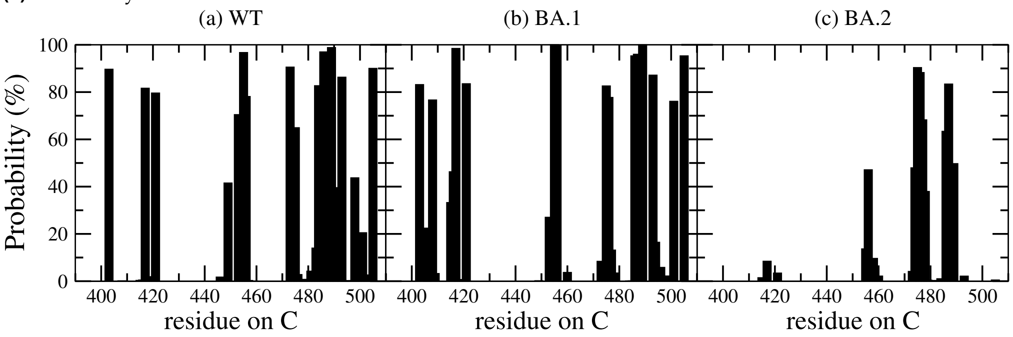

(b) BA.1 to WT

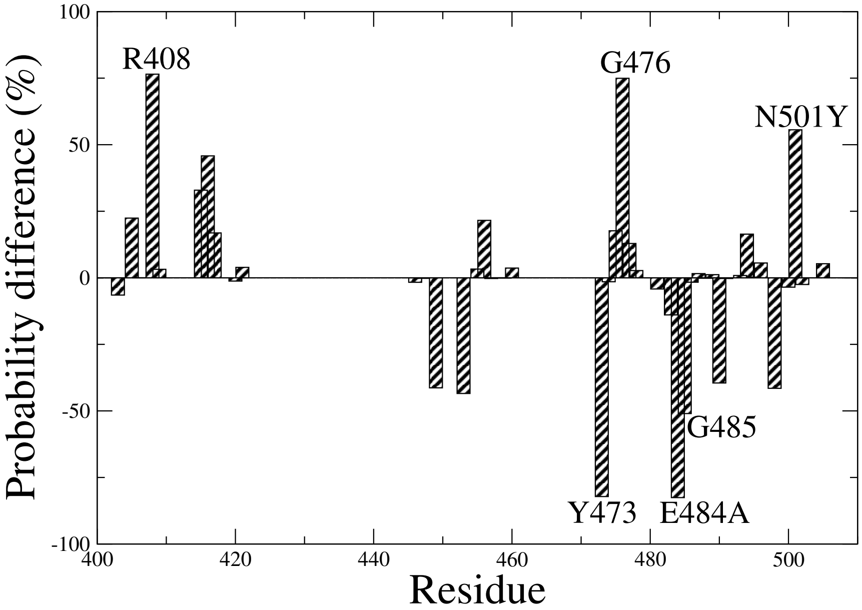

(c) BA.2 to WT

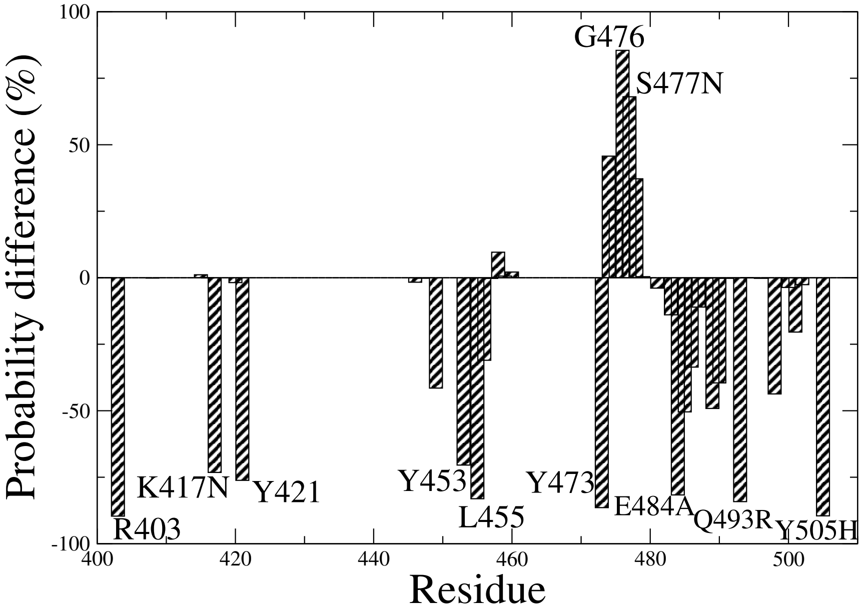

Fig. S11. From RL: (a) Probability of close contacts by residues on RBD-C to RBD-A: clear reduction from WT to BA.1, and further reduction for BA.2, leaving RBD-C most flexible; probability difference of BA.1 (b)/BA.2 (c) to WT. Residues differing by more than 50% are labelled.

(a) Probability of residue contacts

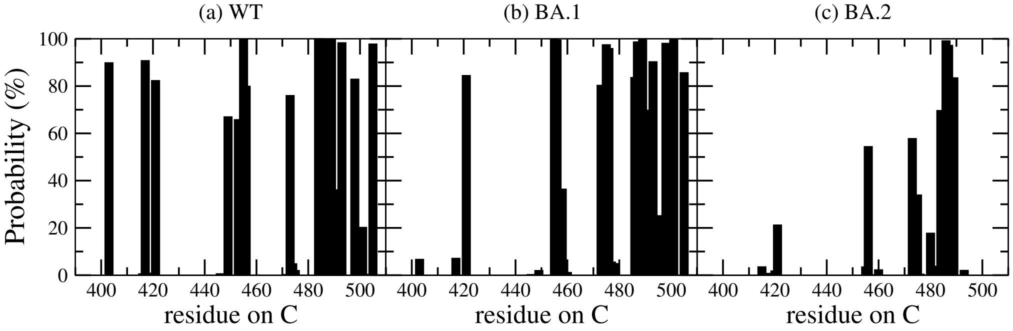

(b) BA.1-WT

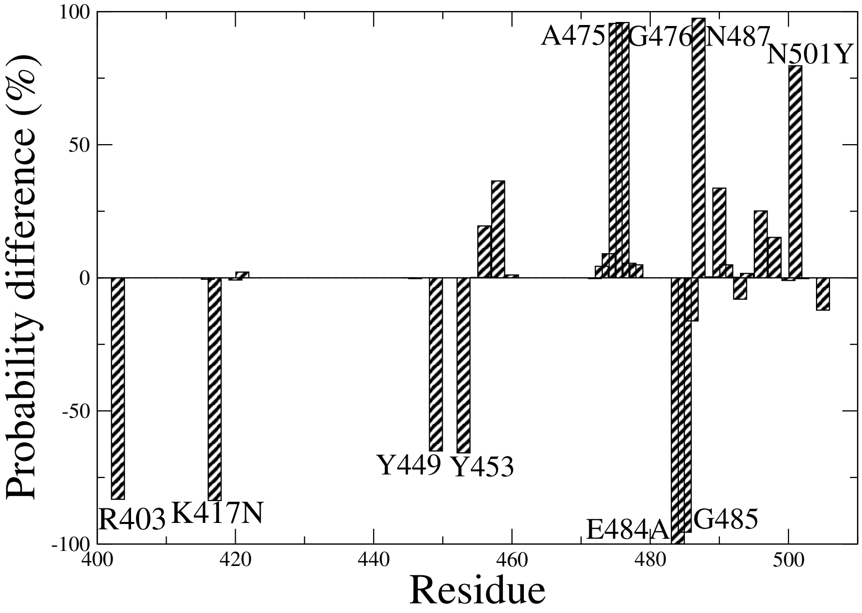

(c) BA.2-WT

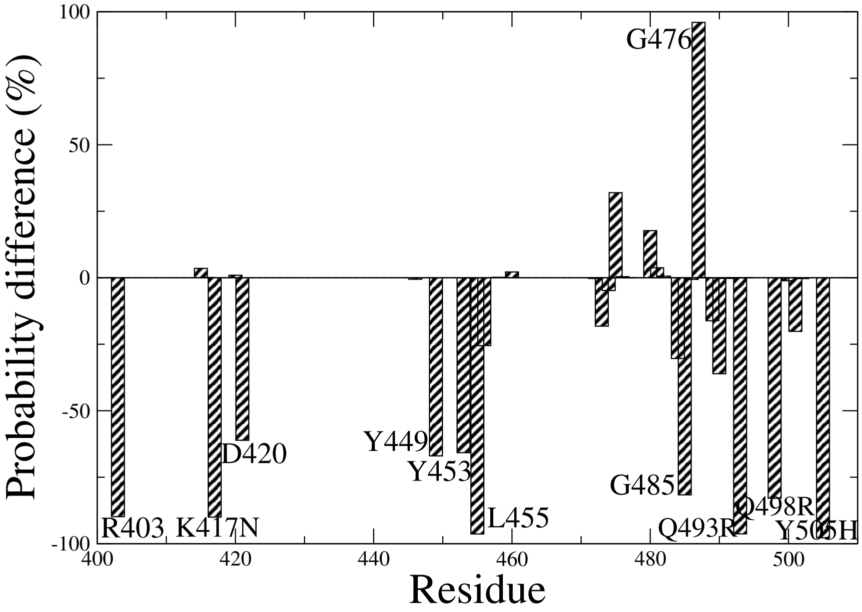

Fig. S12. From RS: (a) Probability of close contacts by residues on RBD-C to RBD-A: clear reduction from WT to BA.1, and further reduction for BA.2, leaving RBD-C most flexible; probability difference of BA.1 (b)/BA.2 (c) to WT. Residues differing by more than 50% are labelled.

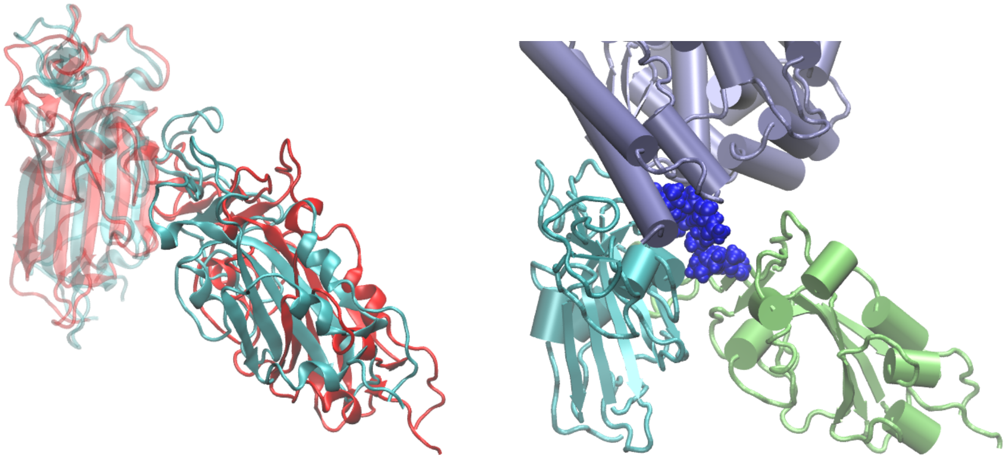

Fig. S13. From RS: (left) Relative positions of RBD-A and RBD-C of BA.1 (cyan) and BA.2 (red). Aligned with RBD-A, it can be seen that RBD-C of BA.2 is further away from its RBD-A than BA.1. (right) Viewing from the inner cavity side, RBD-A and RBD-C are so decoupled that $\bar{N}90_{hACE2}$ (blue spheres) can even occupy the gap in between.

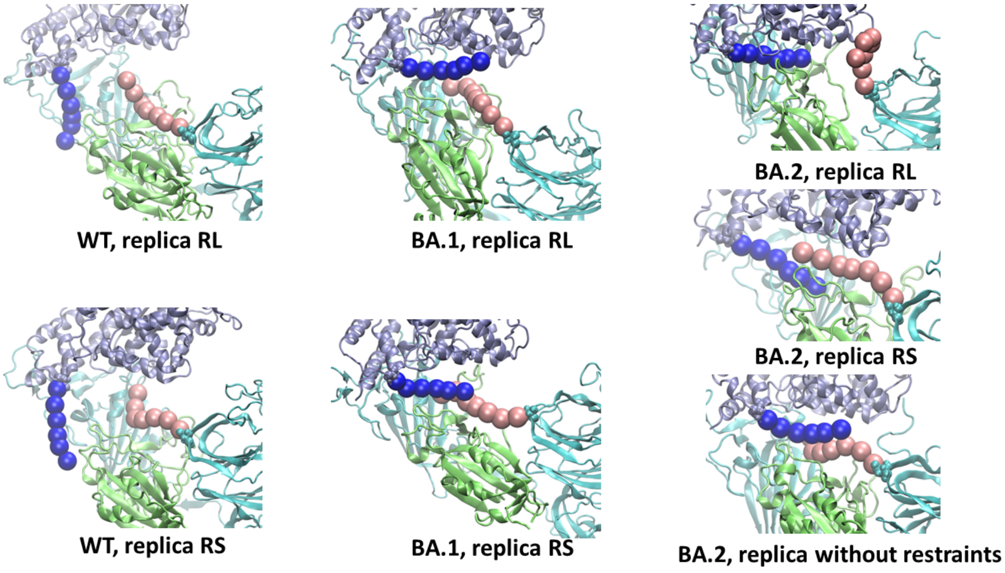

Fig. S14. Side view of $\bar{N}90_{hACE2}$ and $\bar{N}165_{NTD-A}$ at the RBD···hACE2 interface of all replicas: $\bar{N}90_{hACE2}$ is represented by the first carbon atom (C1) of residues 1 to 6 (blue spheres) and $\bar{N}165_{NTD-A}$ by C1 of residues 1 to 7 (pink spheres).

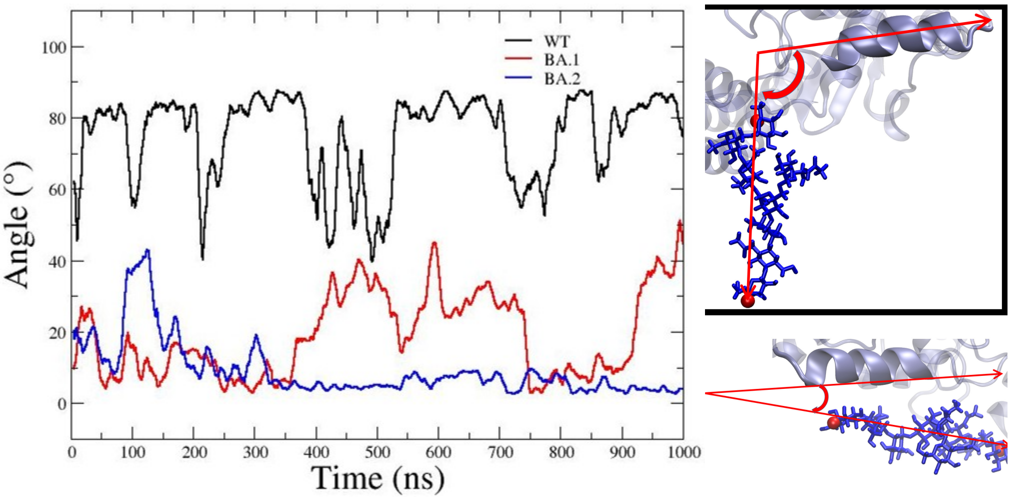

Fig. S15. From RL: Angle between the helix of hACE2 at the interface to S-protein, and the long axis of the glycan on its N90. hACE2 helix with residues 547 to 560 is used, and the long axis of $\bar{N}90_{hACE2}$ is defined by C1 atoms on residues 1 and 6

(a) RL

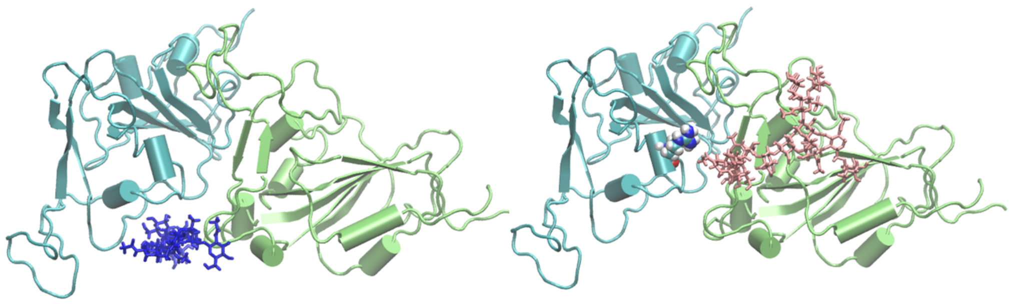

(b) RS

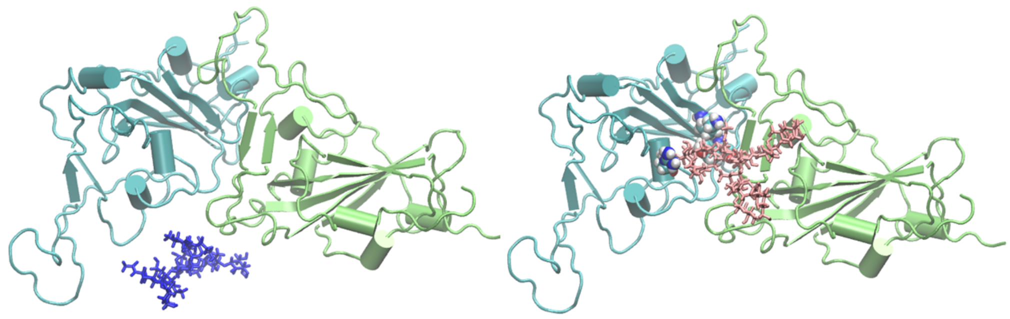

Fig. S16. WT replicas: Top view of the RBD-A and RBD-C to show the positioning of $\bar{N}90_{\mathrm{hACE2}}$ (left) and $\bar{N}165_{\mathrm{NTD-A}}$ (right)

(a) RL

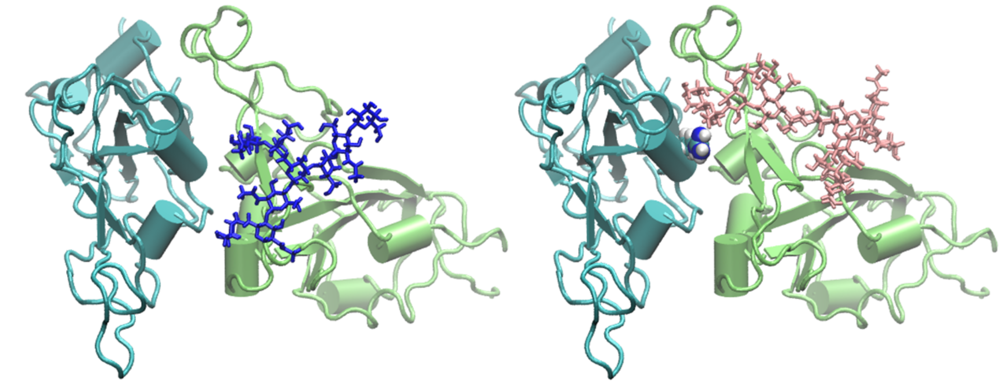

(b) RS

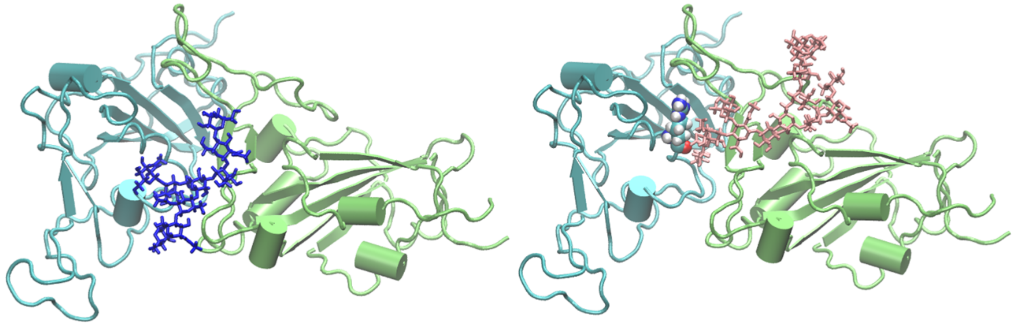

Fig. S17. BA.1 replicas: Top view of the RBD-A and RBD-C to show the positioning of $\bar{N}90_{\mathrm{hACE2}}$(left) and $\bar{N}165_{\mathrm{NTD-A}}$ (right)

(a) RL

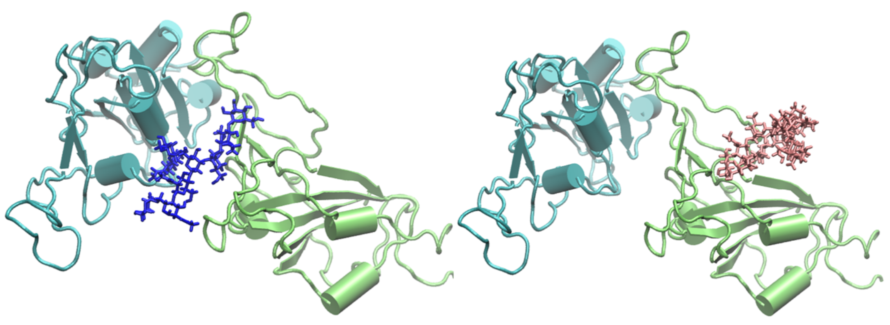

(b) RS

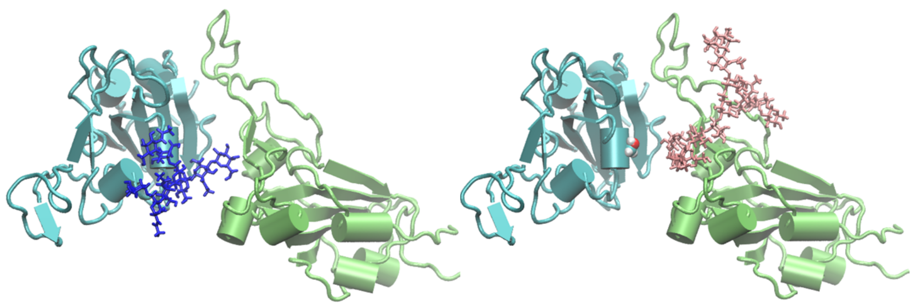

(c) no restraints

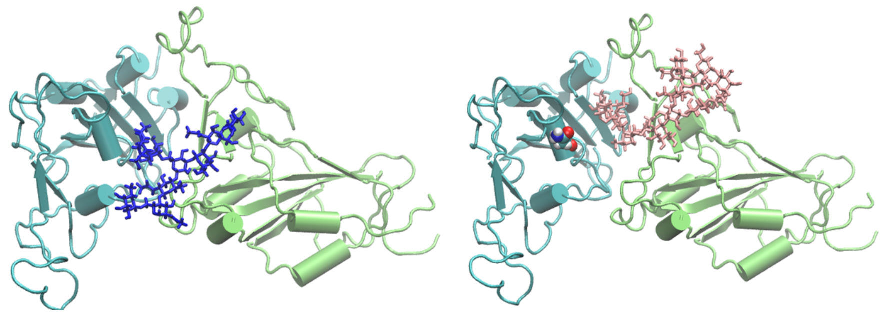

Fig. S18. BA.2 replicas: Top view of the RBD-A and RBD-C to show the positioning of $\bar{N}90_{\mathrm{hACE2}}$ (left) and $\bar{N}165_{\mathrm{NTD-A}}$ (right)
