# Supplementary figures and images for: Structure adaptation in Omicron SARS-CoV-2/hACE2: Biophysical origins of evolutionary driving forces
Source: Biophys J. 2023 Sep 16;122(20):4057–67. doi: 10.1016/j.bpj.2023.09.003 (PMC10624932; doi:10.1016/j.bpj.2023.09.003)

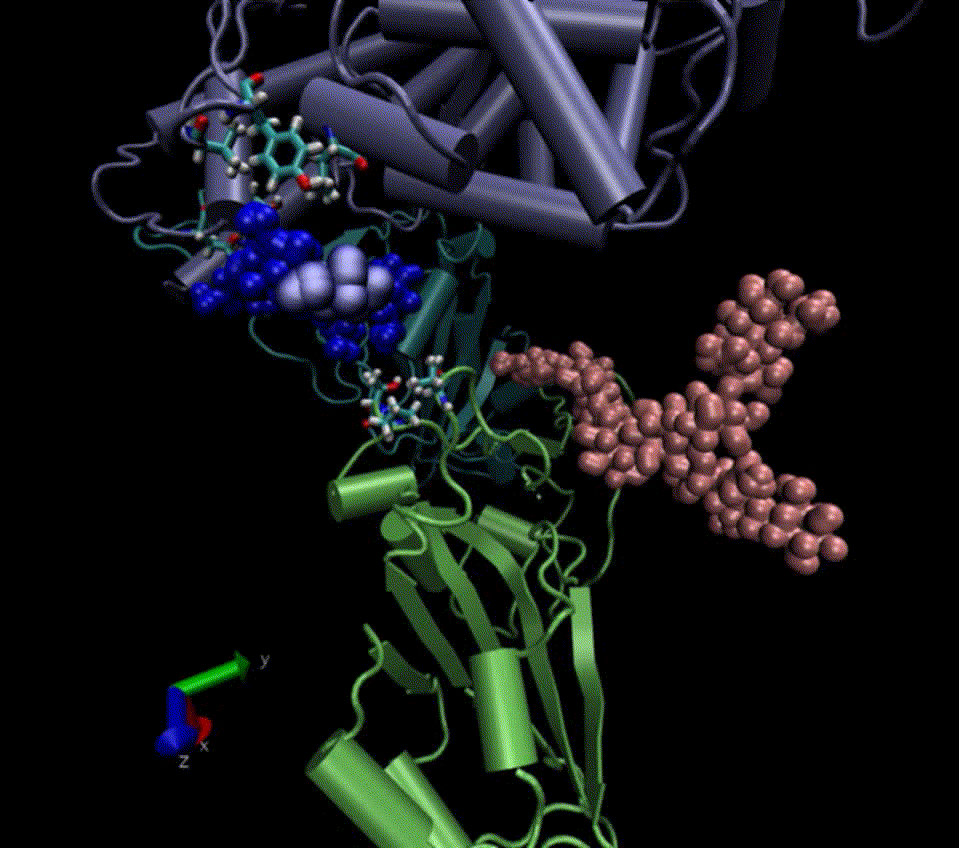

Supplement: Data S3. Video of glycan N90 hACE2 entering the interface between S of BA.2 and hACE2 for RL [file mmc4.zip › BA2-init.gif]
